# Syntheses, structures, and stabilities of aliphatic and aromatic fluorous iodine(I) and iodine(III) compounds: the role of iodine Lewis basicity

**DOI:** 10.3762/bjoc.13.246

**Published:** 2017-11-23

**Authors:** Tathagata Mukherjee, Soumik Biswas, Andreas Ehnbom, Subrata K Ghosh, Ibrahim El-Zoghbi, Nattamai Bhuvanesh, Hassan S Bazzi, John A Gladysz

**Affiliations:** 1 Department of Chemistry, Texas A&M University, P.O. Box 30012, College Station, Texas, 77842-3012, USA,; 2 Department of Chemistry, Texas A&M University at Qatar, P.O. Box 23874, Doha, Qatar

**Keywords:** chlorination, copper mediated perfluoroalkylation, crystal structure, DFT calculations, fluorous, hypervalent iodine, nucleophilic substitution, polar space group

## Abstract

The title molecules are sought in connection with various synthetic applications. The aliphatic fluorous alcohols R_f_*_n_*CH_2_OH (R_f_*_n_* = CF_3_(CF_2_)*_n_*_–1_; *n* = 11, 13, 15) are converted to the triflates R_f_*_n_*CH_2_OTf (Tf_2_O, pyridine; 22–61%) and then to R_f_*_n_*CH_2_I (NaI, acetone; 58–69%). Subsequent reactions with NaOCl/HCl give iodine(III) dichlorides R_f_*_n_*CH_2_ICl_2_ (*n* = 11, 13; 33–81%), which slowly evolve Cl_2_. The ethereal fluorous alcohols CF_3_CF_2_CF_2_O(CF(CF_3_)CF_2_O)*_x_*CF(CF_3_)CH_2_OH (*x* = 2–5) are similarly converted to triflates and then to iodides, but efforts to generate the corresponding dichlorides fail. Substrates lacking a methylene group, R_f_*_n_*I, are also inert, but additions of TMSCl to bis(trifluoroacetates) R_f_*_n_*I(OCOCF_3_)_2_ appear to generate R_f_*_n_*ICl_2_, which rapidly evolve Cl_2_. The aromatic fluorous iodides 1,3-R_f6_C_6_H_4_I, 1,4-R_f6_C_6_H_4_I, and 1,3-R_f10_C_6_H_4_I are prepared from the corresponding diiodides, copper, and R_f_*_n_*I (110–130 °C, 50–60%), and afford quite stable R_f_*_n_*C_6_H_4_ICl_2_ species upon reaction with NaOCl/HCl (80–89%). Iodinations of 1,3-(R_f6_)_2_C_6_H_4_ and 1,3-(R_f8_CH_2_CH_2_)_2_C_6_H_4_ (NIS or I_2_/H_5_IO_6_) give 1,3,5-(R_f6_)_2_C_6_H_3_I and 1,2,4-(R_f8_CH_2_CH_2_)_2_C_6_H_3_I (77–93%). The former, the crystal structure of which is determined, reacts with Cl_2_ to give a 75:25 ArICl_2_/ArI mixture, but partial Cl_2_ evolution occurs upon work-up. The latter gives the easily isolated dichloride 1,2,4-(R_f8_CH_2_CH_2_)_2_C_6_H_3_ICl_2_ (89%). The relative thermodynamic ease of dichlorination of these and other iodine(I) compounds is probed by DFT calculations.

## Introduction

A number of fluorous alkyl iodides, usually of the formula R_f_*_n_*CH_2_CH_2_I or R_f_*_n_*I (R_f_*_n_* = CF_3_(CF_2_)*_n_*_–1_), are commercially available and have seen abundant use as building blocks in fluorous chemistry [1–3]. Fluorous aryl iodides, such as R_f_*_n_*C_6_H_4_I or R_f_*_n_*(CH_2_)*_m_*C_6_H_4_I species, are also often employed as intermediates (typically *m* = 2, 3 and *n* ≥ 6 [1–3]), but only a few have been commercialized [4]. Many research groups have described the syntheses of other types of fluorous alkyl [5–8] and aryl [9–12] iodides [13–17]. The former are ubiquitous by virtue of the large number of perfluoroalkyl iodides R_f_*_n_*I that have been shown to undergo free radical additions to alkenes [7–8].

**Scheme 1:**
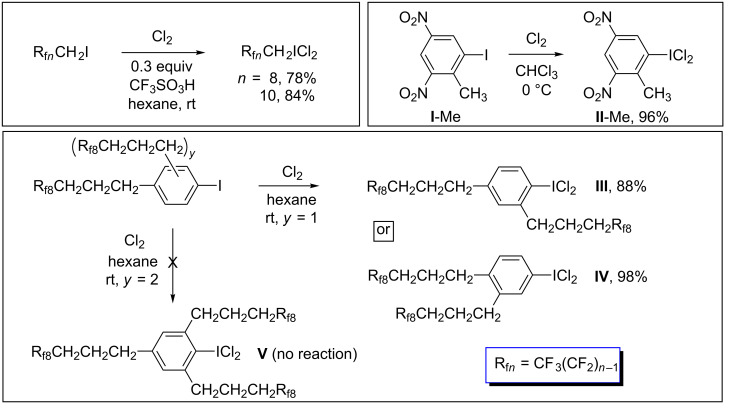
Some previously reported iodine(III) dichlorides relevant to this work.

In previous papers, we have reported convenient preparations of a variety of fluorous alkyl iodides [13–15], aryl iodides [16–17], and hypervalent iodine(III) derivatives [16–19]. The latter have included aliphatic iodine(III) bis(trifluoroacetates) [18–19] and dichlorides [17], and aromatic iodine(III) bis(acetates) [16] and dichlorides [17]. The bis(carboxylates) have been employed as recyclable reagents for oxidations of organic substrates [16,18–19], and some of the dichlorides are depicted in Scheme 1. Others have described additional fluorous iodine(III) species [11,20–22].

Recently, our attention has been directed at two potential applications of iodine containing fluorous compounds. One involves new approaches to phosphorus–carbon bond formation using fluorous alkyl and aryl iodides [23–24]. The other involves the use of fluorous iodine(III) dichlorides for free radical chlorinations [25]. In this regard, phenyl iodine(III) dichloride (PhICl_2_) is an effective free radical chlorinating agent for hydrocarbons [26–27]. Importantly, the mechanism does not involve the liberation of Cl_2_, followed by the textbook sequence of steps. Rather, hydrogen abstraction is effected by a species other than the chlorine radical Cl·, presumably PhICl· [26–27].

One potential attraction of fluorous iodine(III) dichlorides as chlorinating agents would be the recovery and recycling of the fluorous iodide byproduct. Towards this end, higher fluorophilicities are usually advantageous. To a first approximation, these are maximized by increasing the lengths and quantities of the (CF_2_)*_n_* segments, and decreasing the lengths and quantities of any (CH_2_)*_m_* segments [1–3]. However, longer (CF_2_)*_n_* segments are often coupled with lower absolute solubilities [1,28], a logical consequence as one approaches the macromolecular limit of polytetrafluoroethylene. Fluorophilicities are typically quantified by fluorous/organic liquid/liquid phase partition coefficients [1–3]. The most common solvent combination is perfluoro(methylcyclohexane) (CF_3_C_6_F_11_) and toluene.

The objective of this study was to bridge several strategic gaps regarding highly fluorophilic building blocks for the formation of (1) phosphorus–carbon bonded species, and (2) iodine(III) dichloride reagents. For example, aliphatic species of the formula R_f_*_n_*CH_2_CH_2_ICl_2_ are unstable [17]. However, analogs with one less methylene group, R_f_*_n_*CH_2_ICl_2_, have been isolated for *n* = 8 and 10 as depicted in Scheme 1 [17,20]. Although partition coefficients are not available for the iodide R_f8_CH_2_I (the byproduct that would form in most chlorination reactions), they would fall between those of R_f8_I (88.5:11.5 for CF_3_C_6_F_11_/toluene [29]) and R_f8_(CH_2_)_3_I (50.7: 49.3 [30]). These rather modest fluorophilicities would presumably be lower for the more polar dichlorides R_f_*_n_*CH_2_ICl_2_ – a possible disadvantage for reactions in fluorous solvents. In any case, higher homologs that would have more biased partition coefficients were sought.

In the same vein, literature data prompted interest in certain fluorous aromatic iodine(III) dichlorides. For example, the non-fluorous iodine(III) dichloride **II**-Me (Scheme 1) [31], which features two strongly electron-withdrawing nitro groups and a mildly electron-donating methyl group, is easily isolated in analytically pure form from the reaction of the corresponding aryl iodide and Cl_2_, even though the nitro groups render the iodine atom less Lewis basic and thermodynamically less prone to oxidation. The Hammett σ values associated with CF_2_CF_3_ and CF_2_CF_2_CF_3_ substituents (σ_p_ 0.52; σ_m_ 0.47–0.52 [32]) suggest that R_f_*_n_* groups are less electron withdrawing than nitro groups (σ_p_ 0.81; σ_m_ 0.71). Therefore, similar fluorous compounds of the formula (R_f_*_n_*)_2_C_6_H_3_ICl_2_ were seen as realistic targets. The less fluorophilic homologs **III** and **IV** (Scheme 1), which feature three methylene or CH_2_CH_2_CH_2_ "spacers" that electronically insulate the arene ring from the perfluoroalkyl groups, have been previously isolated [17].

As described below, the pursuit of the preceding objectives has met with both success and some unanticipated speed bumps, for which parallel computational studies have provided valuable insight. Regardless, these efforts have resulted in a number of practical preparations that will soon be utilized in further applications [23], and defined various physical properties and stability limits that are useful guides for future research.

## Results

**Scheme 2:**
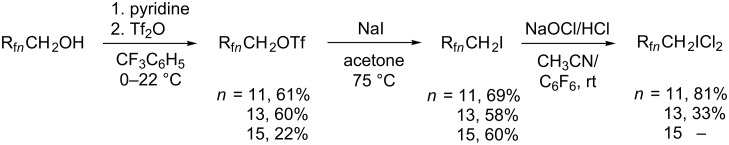
Syntheses of fluorous compounds of the formula R_f_*_n_*CH_2_X.

**Syntheses and reactions, R_f_*_n_*CH_2_I (*n* = 11, 13, 15).** To the authors' knowledge, no fluorous alkyl iodides of the formula R_f_*_n_*CH_2_I are commercially available. Thus, as shown in Scheme 2, a sequence previously employed for lower homologs (*n* = 8, 10 [13]) was investigated. The commercially available alcohols R_f_*_n_*CH_2_OH (*n* = 11, 13, 15) were first converted to the triflates R_f_*_n_*CH_2_OTf using pyridine and triflic anhydride (Tf_2_O) in (trifluoromethyl)benzene (CF_3_C_6_H_5_), an amphoteric solvent that is usually able to dissolve appreciable quantities of both fluorous and non-fluorous solutes [33]. The reactions with *n* = 11 and 13 were conducted at 0 °C, and work-ups gave the expected triflates in 60–61% yields. In contrast, only traces of product were obtained with *n* = 15, presumably due to the poor solubility of the alcohol in virtually any medium. However, the solubilities of fluorous compounds are often highly temperature dependent [28,34], and an analogous reaction at room temperature gave R_f15_CH_2_OTf in 22% yield. The triflates were white solids with some solubility in acetone. They were characterized by IR and NMR (^1^H, ^13^C{^1^H}, ^19^F{^1^H}) spectroscopy and microanalyses as summarized in the experimental section.

The triflates were treated with NaI in acetone at 75 °C. Over the course of 24 h, high conversions to the corresponding fluorous iodides R_f_*_n_*CH_2_I were realized, although at rates much slower than with non-fluorous analogs. Work-ups afforded the products as analytically pure white solids in 58–69% yields, which were characterized analogously to the triflates. All were to some degree soluble in acetone, but as the perfluoroalkyl group lengthened, appropriate cosolvents were required to achieve significant concentrations. In order to obtain ^13^C NMR spectra (*n* = 13, 15), C_6_F_6_ – which is technically a non-fluorous solvent [35] but is nonetheless often effective with fluorous solutes – was employed.

Importantly, these fluorous aliphatic iodides were more fluorophilic than those mentioned in the introduction. Representative partition coefficients were determined as described in the experimental section. Those for R_f15_CH_2_I ranged from >99:<1 for CF_3_C_6_F_11_/toluene to 87:13 for CF_3_C_6_F_11_/acetone. The CF_3_C_6_F_11_/toluene partition coefficient of R_f11_CH_2_I was also >99:<1.

Next, CH_3_CN/C_6_F_6_ solutions of the fluorous aliphatic iodides (*n* = 11, 13) were treated with aqueous NaOCl and conc. HCl. The combination of HCl and a mild oxidant generates Cl_2_, providing a "greener" synthetic approach to iodine(III) dichlorides [36–38]. Accordingly, the target molecules R_f_*_n_*CH_2_ICl_2_ precipitated in 33–81% yields. However, the poor solubilities of these pale yellow powders precluded further purification by the usual protocols. Microanalyses confirmed the presence of chlorine. When ^1^H NMR spectra were recorded in acetone-*d*_6_, new CH_2_ signals 1.37–1.38 ppm downfield of those of the precursors R_f_*_n_*CH_2_I were apparent. However, the NMR samples slowly became greenish yellow, suggestive of dissolved Cl_2_, and the starting iodides were usually evident. The use of Cl_2_ in place of NaOCl/HCl did not give better results.

**Syntheses and reactions, R_fO_*_x_*CH_2_I (*x* = 2–5).** There is an ongoing effort in fluorous chemistry to decrease reliance on perfluorooctyl containing building blocks, which are associated with a variety of environmental issues [39]. One approach is to switch to related ethereal phase tags or "ponytails" [40–41]. Accordingly, oligomeric fluorous ethers that terminate in CH_2_OH groups, CF_3_CF_2_CF_2_O(CF(CF_3_)CF_2_O)*_x_*CF(CF_3_)CH_2_OH, are commercially available. These are abbreviated R_fO_*_x_*CH_2_OH, and the ethereal oxygen atoms have essentially no Lewis base character. In some cases, CF_2_CF_2_OCF(CF_3_)CF_2_OCF(CF_3_)- segments have been found to impart higher fluorophilicites than similar perfluoroalkyl groups [42]. However, the multiple CF(CF_3_) stereocenters are disadvantageous, as they render such compounds mixtures of diastereomers, presenting an impediment to crystallization. In some cases, NMR spectra do not differentiate the diastereomers, and in other cases more complex signal patterns are evident.

**Scheme 3:**
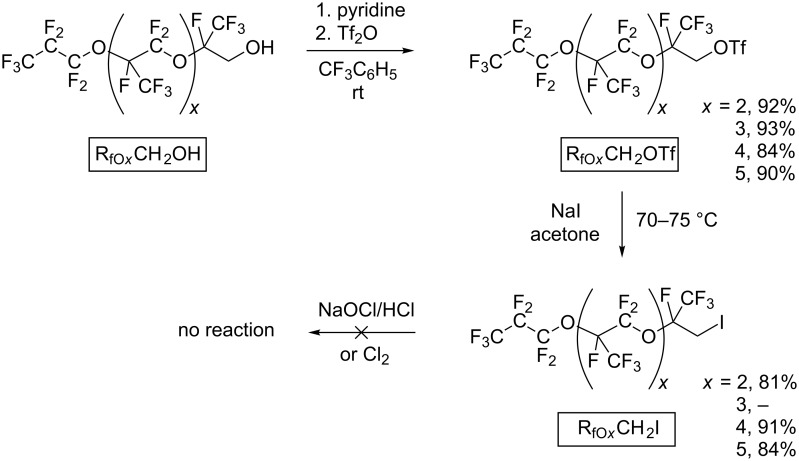
Syntheses of fluorous compounds of the formula CF_3_CF_2_CF_2_O(CF(CF_3_)CF_2_O)*_x_*CF(CF_3_)CH_2_X'.

As shown in Scheme 3, the oligomeric alcohols (*x* = 2–5) were elaborated as described for the non-ethereal alcohols R_f_*_n_*CH_2_OH and a previous report involving the lower non-oligomeric homolog R_fO1_CH_2_OH (*x* = 1 [5]). They were first converted to the triflates R_fO_*_x_*CH_2_OTf using pyridine and triflic anhydride (Tf_2_O). These were soluble in hexane/ethyl acetate and isolated as analytically pure colorless oils in 84–93% yields. Subsequent reactions with NaI in acetone (70–75 °C, *x* = 2,4,5) gave the corresponding iodides R_fO_*_x_*CH_2_I as colorless liquids in 81–91% yields. Unfortunately, efforts to oxidize these compounds to the corresponding iodine(III) dichlorides using the conditions in Scheme 1 and Scheme 2 were unsuccessful. NMR analyses of crude reaction mixtures showed only starting material.

**Attempted syntheses of R_f_*_n_*ICl_2_.** Prior to the efforts described in the previous sections, iodine(III) dichlorides derived from perfluoroalkyl iodides R_f_*_n_*I were considered as targets. Since these lack sp^3^ carbon–hydrogen bonds, they are not susceptible to possible chlorination or other degradation under free radical chlorination conditions. However, no reactions were observed when R_f_*_n_*I were treated with Cl_2_ or NaOCl/HCl.

**Scheme 4:**
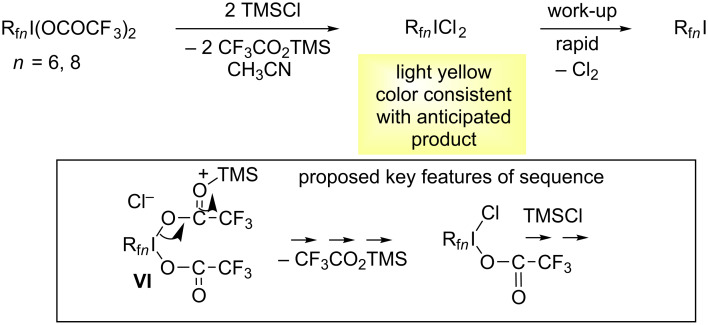
Attempted syntheses of aliphatic fluorous iodine(III) dichlorides R_f_*_n_*ICl_2_.

Nonetheless, perfluoroalkyl iodides R_f_*_n_*I (*n* = 6–8, 10, 12) can be oxidized using various recipes (e.g., 80% H_2_O_2_ in trifluoroacetic acid anhydride) to the iodine(III) bis(trifluoroacetates) R_f_*_n_*I(OCOCF_3_)_2_ in high isolated yields [18–19,21]. It was thought that these might, in turn, react with TMSCl as sketched in Scheme 4 to provide "back door" entries to the target compounds R_f_*_n_*ICl_2_. Indeed, when these reactions were carried out, the samples exhibited the appropriate characteristic bright yellow colors (*n* = 6, 8). However, upon work-up only the original perfluoroalkyl iodides R_f_*_n_*I were isolated. Hence, it is concluded that the target compounds are thermodynamically and kinetically unstable with respect to Cl_2_ elimination, consistent with the failure of the direct reaction and a lower Lewis basicity of the iodine atom as compared to R_f_*_n_*CH_2_I.

After these experiments were carried out, we became aware of the isolation of CF_3_ICl_2_ (R_f1_ICl_2_) from the reaction of CF_3_IClF and TMSCl at −40 °C [43]. This route is conceptually similar to that shown in Scheme 4, and a crystal structure of CF_3_ICl_2_ could even be obtained. However, consistent with our observations, the compound decomposed above −35 °C.

**Syntheses and reactions, aryl iodides with one perfluoroalkyl group.** Aromatic compounds are challenging to render highly fluorophilic [16,30,44]. For example, the singly-phase-tagged arene C_6_H_5_CH_2_CH_2_CH_2_R_f8_ gives a 49.5:50.5 CF_3_C_6_F_11_/toluene partition coefficient. Values for doubly tagged analogs fall into the range (90.7–91.2):(9.3–8.8) (*o*, *m, p*-isomers), and that for the triply tagged species 1,3,5-C_6_H_3_(CH_2_CH_2_CH_2_R_f8_)_3_ is >99.7:<0.3 [30]. As noted above, longer perfluoroalkyl segments increase fluorophilicities, as do shorter methylene segments (compare the partition coefficients of C_6_H_5_R_f8_ (77.5:22.5) or 1,4-(R_f8_)_2_C_6_H_4_ (99.3:0.7) with the preceding examples [29]). Thus, in considering various fluorous aryliodine(III) dichloride targets, initial efforts were directed at systems with at least two R_f_*_n_* substituents per arene ring. Given the ready isolation of the dinitro-substituted aryliodine(III) dichloride **II**-Me in Scheme 1 [31], this was seen as a surefire objective.

**Scheme 5:**
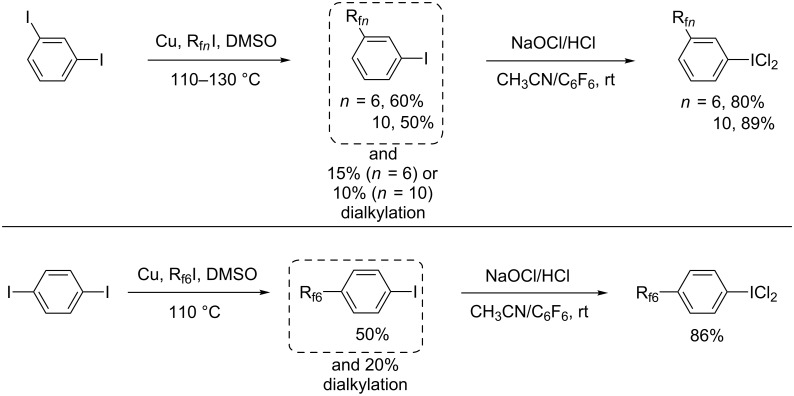
Syntheses of aromatic fluorous compounds with one perfluoroalkyl group.

However, this was not to be, so the results in this and the following section are presented in inverse chronological order, focusing first on arenes with one R_f_*_n_* substituent. As shown in Scheme 5 (top), the commercially available *meta* diiodide 1,3-C_6_H_4_I_2_ was treated with copper (1.0 equiv) and R_f6_I (0.5 equiv; a deficiency to help suppress dialkylation) in DMSO at 110 °C. Similar recipes have previously been used to couple aryl iodides and R_f_*_n_*I building blocks [45–46]. Work-up gave the target compound 1,3-R_f6_C_6_H_4_I in 60% yield (based upon limiting R_f6_I). Some of the previously reported dialkylation product 1,3-(R_f6_)_2_C_6_H_4_ was also formed [47–48], but was easily separated due to its differential fluorophilicity (extraction of a CH_3_CN solution with perfluorohexane). An analogous procedure with R_f10_I gave the higher homolog 1,3-R_f10_C_6_H_4_I in 50% yield [9], and a lesser amount of what was presumed to be the dialkylation product. The analogous *para* diiodide 1,4-C_6_H_4_I_2_ gave parallel chemistry, as illustrated by the reaction with R_f6_I to give 1,4-R_f6_C_6_H_4_I (50% [4,12]) in Scheme 5 (bottom).

The three aryl iodides R_f_*_n_*C_6_H_4_I thus obtained were treated with NaOCl/HCl per the sequence in Scheme 2. As shown in Scheme 5, work-ups gave the corresponding iodine(III) dichlorides R_f_*_n_*C_6_H_4_ICl_2_ as pale yellow powders in 80–89% yields. Although these were clean by NMR, only one gave a correct microanalysis. As illustrated in Figure S1 (Supporting Information File 1), CDCl_3_/C_6_F_6_ solutions of 1,3-R_f6_C_6_H_4_ICl_2_ and 1,4-R_f6_C_6_H_4_ICl_2_ containing an internal standard were monitored by ^1^H NMR. Slow partial evolution of Cl_2_ to give the iodides 1,3-R_f6_C_6_H_4_I and 1,4-R_f6_C_6_H_4_I was observed (7% and 27% conversion over 60 h, respectively).

**Scheme 6:**
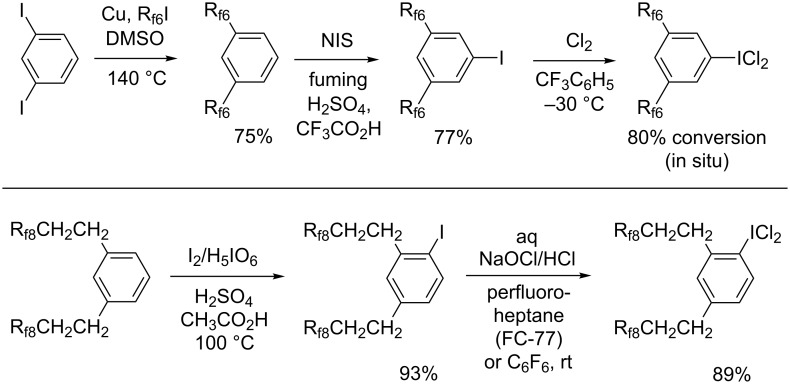
Syntheses of aromatic fluorous compounds with two perfluoroalkyl groups.

**Syntheses and reactions, aryl iodides with two perfluoroalkyl groups.** In a previously reported procedure [48], the diiodide 1,3-C_6_H_4_I_2_ was treated with copper (5.1 equiv) and R_f6_I (2.2 equiv) in DMSO at 140 °C. As shown in Scheme 6 (top), the bis(perfluorohexyl) adduct 1,3-(R_f6_)_2_C_6_H_4_, which was the undesired byproduct in Scheme 5 (top), was isolated in 75% yield. Subsequent iodination using NIS in fuming H_2_SO_4_/CF_3_CO_2_H afforded the "all *meta*" iodide 1,3,5-(R_f6_)_2_C_6_H_3_I in 77% yield. The substitution pattern was evident from the ^1^H NMR spectrum.

**Figure 1:**
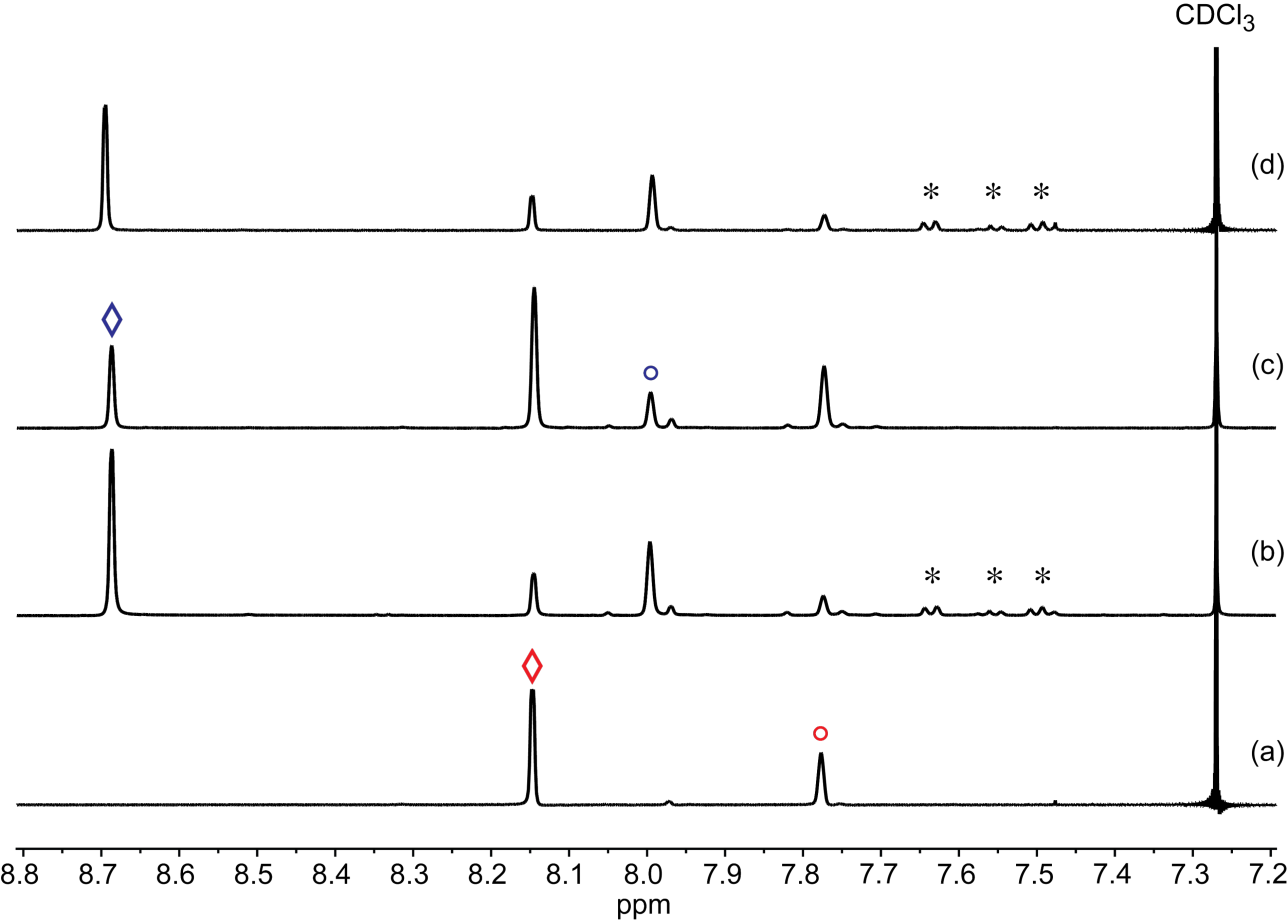
Partial ^1^H NMR spectra (sp^2^ CH, 500 MHz, CDCl_3_) relating to the reaction of 1,3,5-(R_f6_)_2_C_6_H_3_I and Cl_2_ in CF_3_C_6_H_5_ at −30 to −35 °C: (a) starting 1,3,5-(R_f6_)_2_C_6_H_3_I; (b) aliquot taken after 24 h and removing nearly all CF_3_C_6_H_5_ (* = residual signals) by rotary evaporation (75:25 ArICl_2_/ArI); (c) aliquot taken after 24 h and removing all solvents by oil pump vacuum at −40 °C (35:65 ArICl_2_/ArI); (d) the sample from b, which was redissolved in CF_3_C_6_H_5_, kept at −35 °C for 7 d, and worked up as in b (65:35 ArICl_2_/ArI). The signals for the protons *para* and *ortho* to the iodine atoms are denoted ° and ◊ (red = ArI; blue = ArICl_2_).

When Cl_2_ gas was sparged through a CF_3_C_6_H_5_ solution of 1,3,5-(R_f6_)_2_C_6_H_3_I at −30 °C to −35 °C, the sample turned bright yellow. Two aliquots were removed. The ^1^H NMR spectrum of one (Figure 1b) showed two downfield shifted signals (cf. Figure 1a), which were attributed to the target molecule 1,3,5-(R_f6_)_2_C_6_H_3_ICl_2_. Integration indicated 77:23 and 75:25 ArICl_2_/ArI ratios prior to and after solvent removal (room temperature, rotary evaporation). The isolated material was redissolved in CF_3_C_6_H_5_ and kept at −35 °C. After 7 d, the solvent was again removed by rotary evaporation, giving a 65:35 ArICl_2_/ArI mixture (Figure 1d). The solvent was removed from the second aliquot by oil pump vacuum at −40 °C. This gave a 35:65 ArICl_2_/ArI mixture as a pale white solid (Figure 1c). A variety of attempts to achieve higher conversions or isolate pure 1,3,5-(R_f6_)_2_C_6_H_3_ICl_2_ were unsuccessful. It was concluded that 1,3,5-(R_f6_)_2_C_6_H_3_ICl_2_ was much more labile with respect to Cl_2_ evolution than the fluorous aryliodine(III) dichlorides shown in Scheme 5, and that the 75–80% conversions reflected a thermodynamic limit.

Next, analogs of 1,3,5-(R_f6_)_2_C_6_H_3_ICl_2_ with less electron-deficient iodine atoms were sought. As shown in Scheme 1, related fluorous aryliodine(III) dichlorides with three-methylene spacers, (R_f8_CH_2_CH_2_CH_2_)_2_C_6_H_3_ICl_2_, had been isolated (the isomers **III**, **IV**) [17]. Recently, a potential precursor with two-methylene spacers, 1,3-(R_f8_CH_2_CH_2_)_2_C_6_H_4_, became readily available [49]. Accordingly, it could be iodinated with I_2_/H_5_IO_6_ as shown in Scheme 6 (bottom) to give 1,2,4-(R_f8_CH_2_CH_2_)_2_C_6_H_3_I [50] in 93% yield after work-up. The ^1^H NMR spectrum clearly indicated the regioisomer in which the iodide is *ortho* and *para* to the two alkyl substituents. This contrasts with the iodination of 1,3-(R_f6_)_2_C_6_H_4_, in which the substituents function as *meta* directing groups.

As shown in Scheme 6, reactions of C_6_F_6_ or perfluoroheptane solutions of 1,2,4-(R_f8_CH_2_CH_2_)_2_C_6_H_3_I and NaOCl/HCl gave the corresponding iodine(III) dichloride 1,2,4-(R_f8_CH_2_CH_2_)_2_C_6_H_3_ICl_2_ [50] as a white powder in 89% yield. This material was stable at room temperature and gave a microanalysis consistent with a monohydrate. Hence, the iodine atom in benzenoid compounds with two R_f8_CH_2_CH_2_ substituents is sufficiently Lewis basic to support a dichloride, but analogs with two R_f6_ substituents are not.

**Figure 2:**
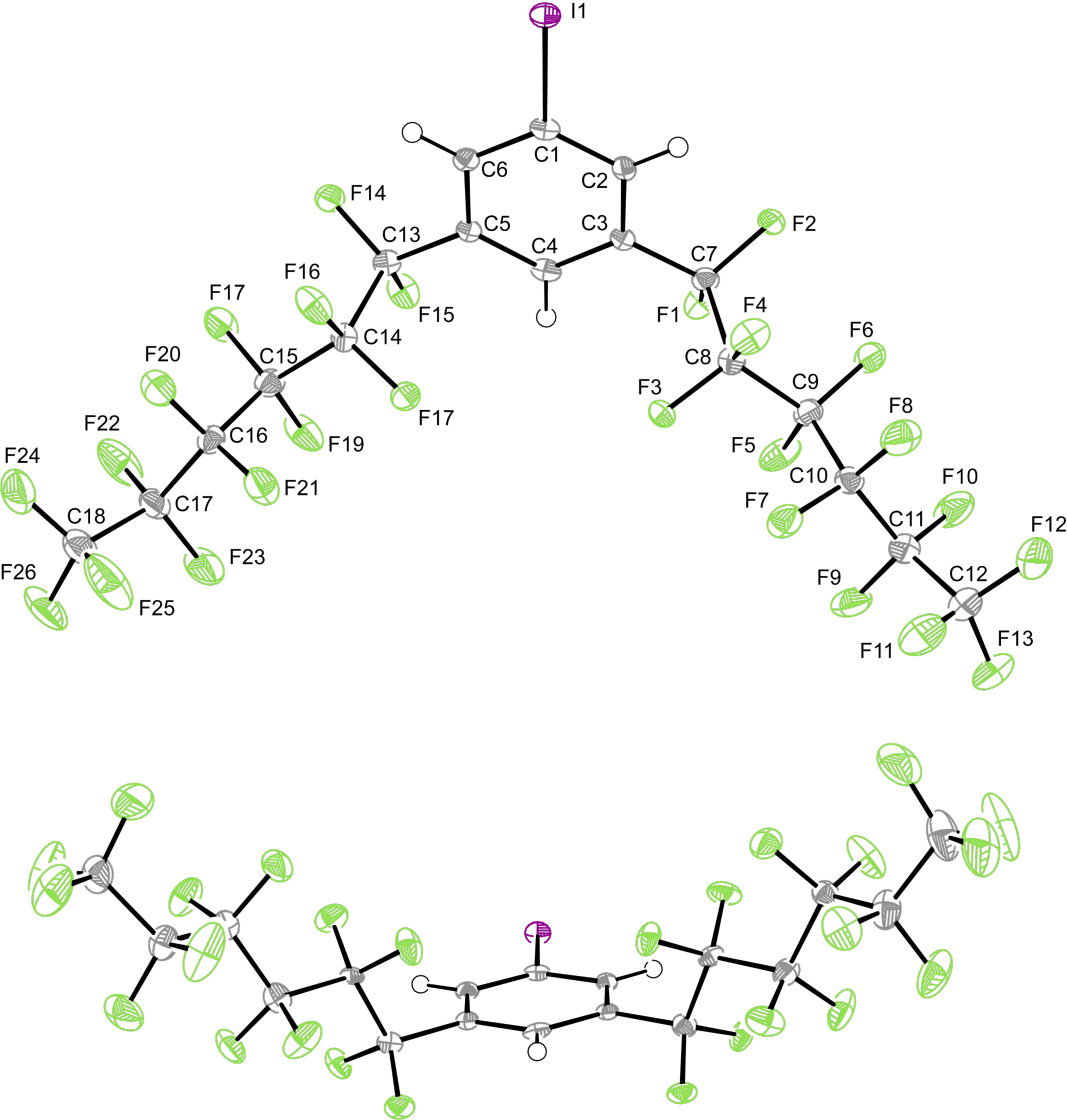
Two views of the molecular structure of 1,3,5-(R_f6_)_2_C_6_H_3_I with thermal ellipsoids at the 50% probability level. Key bond lengths (Å) and angles (°): C1–I1 2.099(3), C1–C2 1.391(4), C2–C3 1.386(4), C3–C4 1.393(4), C4–C5 1.387(4), C5–C6 1.393(4), C6–C1 1.394(4), C3–C7 1.501(4), C5–C13 1.508(4), average of 10 CF–CF 1.545(5), I1–C1–C2 119.0(2), C1–C2–C3 118.8(3), C2–C3–C4 121.0(3), C3–C4–C5 119.2(3), C4–C5–C6 121.1(3), C5–C6–C1 118.5(3), C6–C1–C2 121.4(3), C6–C1–I1 119.5(2), C2–C3–C7 119.7(2), C4–C3–C7 119.3(2), C4–C5–C13 119.6(2), C6–C5–C13 119.3(2), average of 8 CF–CF–CF 115.0(10).

**Figure 3:**
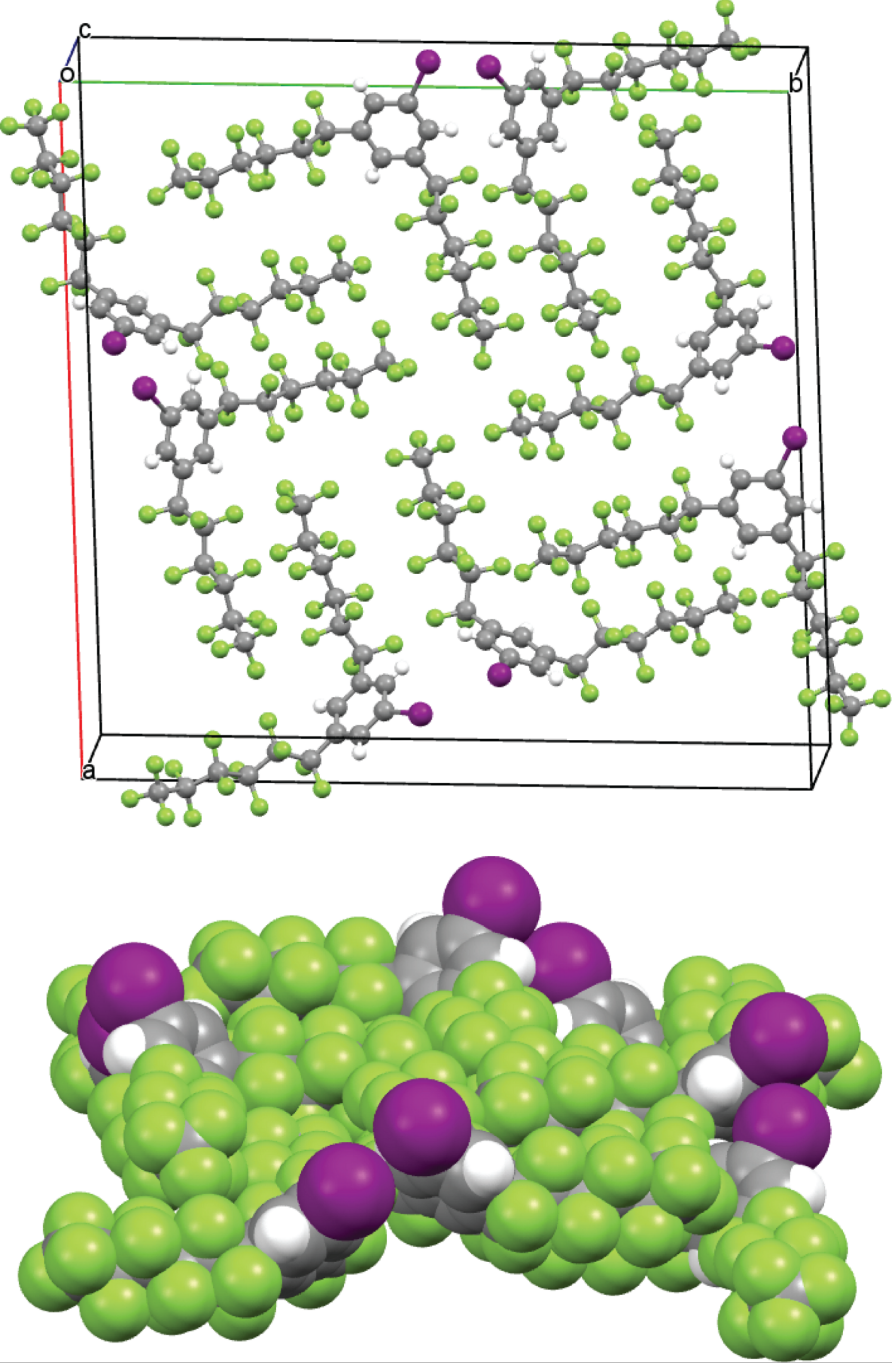
Ball-and-stick and space filling representations of the unit cell of 1,3,5-(R_f6_)_2_C_6_H_3_I.

**Structural and computational data.** Crystal structures of fluorous compounds were virtually unknown 20 years ago [51], so opportunities to acquire structural data are usually seized. Crystals of 1,3,5-(R_f6_)_2_C_6_H_3_I could be grown as described in the experimental section. X-ray data were collected, and the structure determined, as summarized in Table 1 and the experimental section. Two views of the molecular structure and key metrical parameters are provided in Figure 2. Two perspectives of the unit cell (*Z* = 8) are provided in Figure 3. There are some unusual features associated with the packing and space group, and these are treated in the discussion section.

**Table 1:** Summary of crystallographic data for 1,3,5-(R_f6_)_2_C_6_H_3_I.

empirical formula	C_18_H_3_F_26_I
formula weight	840.10
diffractometer	Bruker GADDS X-ray (three-circle)
temperature [K]	110(2)
wavelength [Å]	1.54178
crystal system	tetragonal
space group	*I*4
unit cell dimensions	
*a* [Å]	29.6474(9)
*b* [Å]	29.6474(9)
*c* [Å]	5.5976(2)
α [°]	90
β [°]	90
γ [°]	90
*V* [Å^3^]	4920.1(3)
*Z*	8
ρ_calcd_ [Mg/m^3^]	2.268
µ [mm^−1^]	12.238
F(000)	3184
crystal size [mm^3^]	0.40 × 0.02 × 0.02
θ limit [°]	2.11 to 59.94
index ranges [*h*, *k*, *l*]	−33, 32; −33, 33; −6, 5
reflections collected	53384
independent reflections	3568
*R*(int)	0.0540
completeness (%) to θ (°)	99.8 (59.94)
max. and min. transmission	0.7919 and 0.0843
data/restraints/parameters	3568/1/407
goodness-of-fit on *F*^2^	0.991
*R* indices (final) [*I* > 2σ(*I*)]	
*R*_1_	0.0156
w*R*_2_	0.0355
*R* indices (all data)	
*R*_1_	0.0172
*wR*_2_	0.0357
largest diff. peak and hole [eÅ^−3^]	0.227 and −0.532

In order to help interpret the accessibilities and/or stabilities of the various iodine(III) dichlorides described above, the gas phase free energies of chlorination

**Figure 4:**
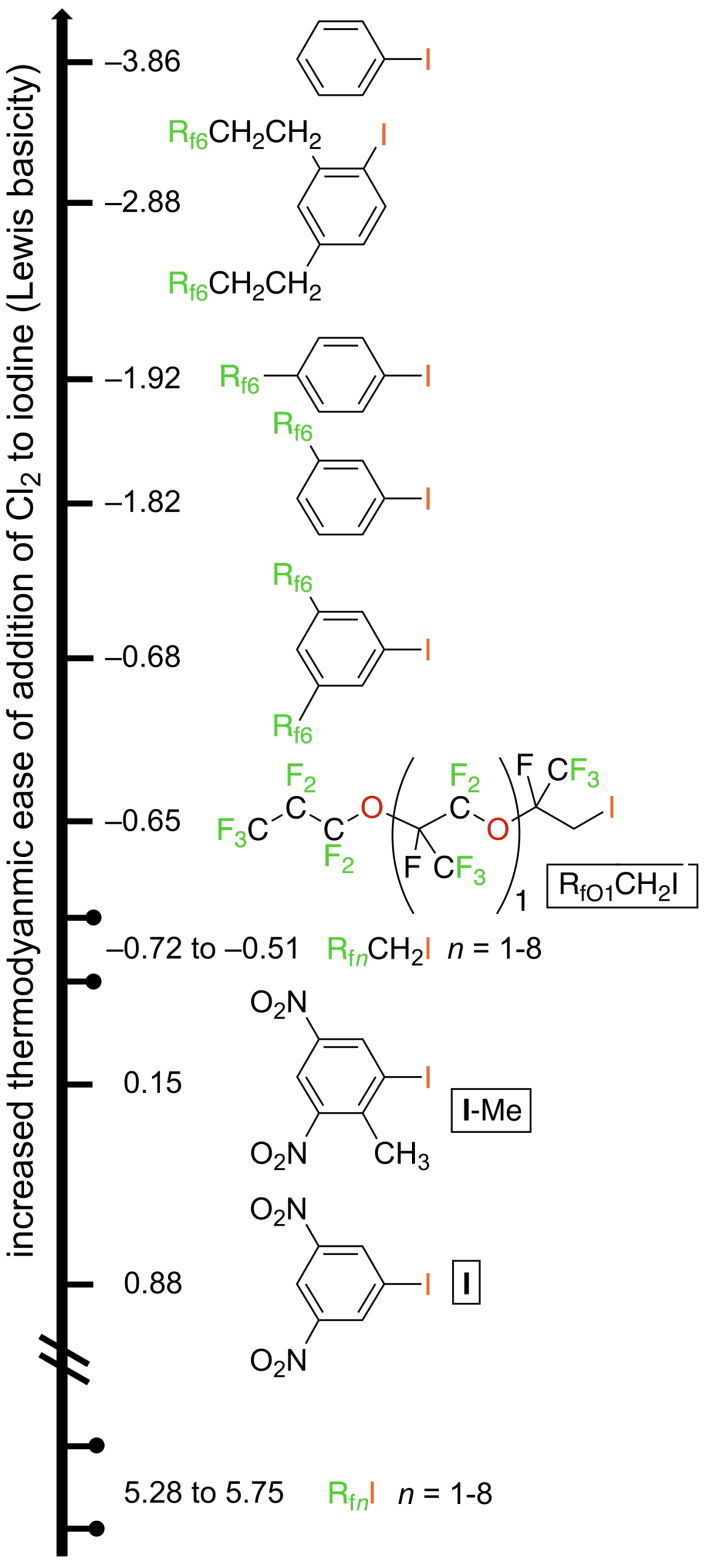
Free energies of chlorination of relevant aryl and alkyl iodides to the corresponding iodine(III) dichlorides in the gas phase (kcal/mol), presented in a ladder format (each iodide is more Lewis basic than that shown below it).

[1]
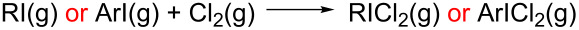


were computed by DFT methods as described in the experimental section and summarized in Table S1 (Supporting Information File 1). The data are presented in "ladder format" in Figure 4, with the substrates that undergo more exergonic chlorinations placed higher. The energy difference between any pair of compounds is equal to that expressed by the corresponding isodesmic equation:

[2]
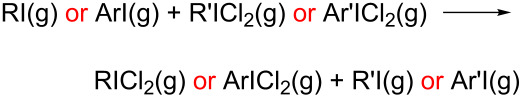


The validity of the data was supported by the good agreement of the computed structure of 1,3,5-(R_f6_)_2_C_6_H_3_I with the crystal structure (Figure 2). An overlay, provided in Figure S2 (Supporting Information File 1), shows only very slightly increasing conformational differences as the perfluorohexyl groups extend from the arene. For the aliphatic compounds (R_f_*_n_*I, R_f_*_n_*CH_2_I, R_fO_*_x_*CH_2_ICl_2_), the free energies of chlorination were calculated for a series of chain lengths. As summarized in Figure 4 and tabulated in Table S1 (Supporting Information File 1), the Δ*G* values within each series varied by less than 0.5 kcal/mol. In all cases, vertical ionization potentials (not presented) followed analogous trends.

The iodine(III) dichlorides formed in the more exergonic reactions (upper portion of Figure 4) would be expected to be more stable with respect to Cl_2_ evolution. Thus, the data are consistent with the stability order R_f_*_n_*CH_2_ICl_2_ >> R_f_*_n_*ICl_2_ evident from Scheme 2 and Scheme 4. However, they also imply that the ethereal systems R_fO_*_x_*CH_2_ICl_2_ (Scheme 3; −0.65 kcal/mol, *x* = 1) should be more stable than R_f_*_n_*CH_2_ICl_2_ (−0.53 to −0.59 kcal/mol, *n* = 4–8). All attempts to generate the former have been unsuccessful to date. Hence, there is either a kinetic barrier to the formation of R_fO_*_x_*CH_2_ICl_2_ that is not overcome under the conditions of Scheme 3, or an unrecognized, presumably non-electronic, destabilizing feature.

In the same vein, there must be a mitigating factor, such as solubility, that allows the isolation of the dinitro-substituted aryliodine(III) dichloride **II**-Me in pure form (Scheme 1), but not the bis(perfluorohexyl) species 1,3,5-(R_f6_)_2_C_6_H_3_ICl_2_ (Scheme 6, Figure 1). The latter is derived from a more Lewis basic aryl iodide, with Cl_2_ addition 0.83 kcal/mol more favorable. The *ortho* methyl group in **II**-Me plays a moderately stabilizing role, with Cl_2_ addition to **I**-Me 0.73 kcal/mol more favorable than **I**. Otherwise, the computations (carried out with R_f6_ groups to aid comparability) nicely predict the relative stabilities of the fluorous aryliodine(III) dichlorides (1,2,4-(R_f8_CH_2_CH_2_)_2_C_6_H_3_ICl_2_ [50] > 1,4-R_f6_C_6_H_4_ICl_2_ > 1,3-R_f6_C_6_H_4_ICl_2_ > 1,3,5-(R_f6_)_2_C_6_H_3_ICl_2_).

## Discussion

The preceding experimental data define the stability limits associated with a broad range of fluorous aliphatic and aromatic iodine(III) dichlorides. Aliphatic compounds of the formula R_f_*_n_*ICl_2_ are clearly very unstable with respect to Cl_2_ loss, although there is literature precedent for their synthesis and isolation from other iodine(III) precursors under exacting low temperature conditions [43]. When an insulating methylene group is introduced between the fluorous moiety and the ICl_2_ group, the situation improves. Compounds of the formula R_f_*_n_*CH_2_ICl_2_ can generally be isolated, although they are somewhat labile towards Cl_2_ loss. In contrast, efforts to prepare the ethereal systems R_fO_*_x_*CH_2_ICl_2_ by the chlorination of R_fO_*_x_*CH_2_I have been unsuccessful. This poses a conundrum with respect to the DFT calculations; they seemingly possess sufficient Lewis basicity (Figure 4), but there appears to be a kinetic barrier.

In contrast, fluorous aromatic iodine(III) dichlorides bearing a single perfluoroalkyl group, R_f_*_n_*C_6_H_4_ICl_2_, are easily isolated in analytically pure form (Scheme 5), although they are still subject to slow Cl_2_ loss in solution (Figure S1, Supporting Information File 1). However, it has not yet proved possible to quantitatively generate analogs with two perfluoroalkyl groups by chlorinations of iodine(I) precursors (Scheme 6, top); 75–80% conversions are the maximum realized to date. In contrast, chlorinations of the doubly substituted substrates (R_f_*_n_*(CH_2_)*_m_*)_2_C_6_H_3_I (*m* = 2, 3) go to completion, as exemplified in Scheme 1 (bottom) and Scheme 6 (bottom). The intervening methylene groups partially insulate the iodine atoms from the electron-withdrawing perfluoroalkyl segments, enhancing Lewis basicities.

**Scheme 7:**
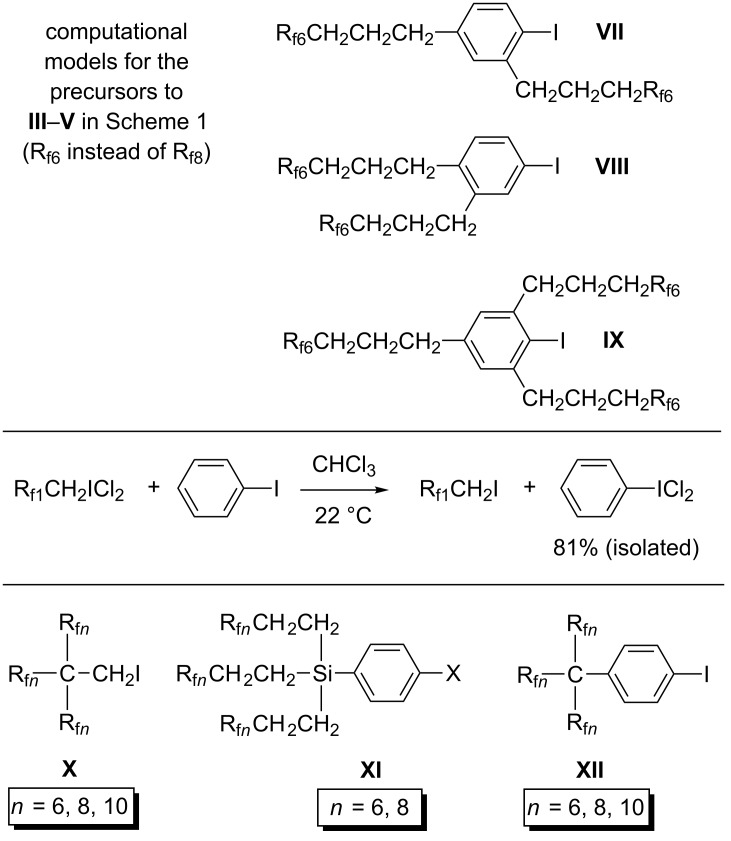
Other relevant fluorous compounds and reactions.

However, it has not yet proved possible to access related compounds with three R_f_*_n_*(CH_2_)*_m_* groups, at least when two of them are *ortho* to the iodine atom, as exemplified by **V** in Scheme 1 [17]. To probe this point, the DFT calculations were extended to the R_f6_ homologs of the precursors of the three aryliodine(III) dichlorides in Scheme 1. These correspond to **VII**, **VIII**, and **IX** in Scheme 7 (top). The Δ*G* values obtained were −3.40, −3.75, and −4.15 kcal/mol, respectively. Thus, the third R_f_*_n_*(CH_2_)_3_ substituent enhances the exergonicity of Cl_2_ addition. Hence, the failure to observe a reaction must represent a kinetic phenomenon. A second "ladder", augmented with the additional alkyl and aryl iodides analyzed in the discussion section, is provided in Figure S3 (Supporting Information File 1).

Interestingly, isodesmic reactions corresponding to Equation 2 in the preceding section can actually be carried out. For example, Cl_2_ can be transferred from the fluorous aliphatic iodine(III) dichloride R_f1_CH_2_ICl_2_ (CF_3_CH_2_ICl_2_) to phenyl iodide as shown in Scheme 7 (middle) [52]. The Δ*G* value for the addition of Cl_2_ to phenyl iodide is computed to be −3.86 kcal/mol, as compared to −0.72 kcal/mol for R_f1_CH_2_I. Curiously, the introduction of two R_f_*_n_*(CH_2_)_3_ substituents that are *ortho*/*para* or *meta*/*para* to iodine is thermodynamically deactivating for Cl_2_ addition (**VII**/**III** and **VIII**/**IV**; −3.40 to −3.75 kcal/mol), whereas the introduction of three that are *ortho*/*para*/*ortho* is activating (**IX**/**V**; −4.15 kcal/mol) but kinetically inhibiting for steric reasons.

As noted in the introduction, a long-standing goal has been to realize highly fluorophilic aliphatic and aromatic iodine(I) compounds and iodine(III) dichlorides. The preceding results raise the question, "*quo vadis*?" When the compounds R_f_*_n_*CH_2_I and R_f_*_n_*CH_2_ICl_2_ reach *n* = 15 (Scheme 2), they are close to approaching a practical solubility limit, although the former gives a highly biased CF_3_C_6_F_11_/toluene liquid/liquid partition coefficient. Branched analogs may be more tractable. However, DFT calculations show that chlorinations of substrates such as (R_f_*_n_*)_3_CCH_2_I **X** (Scheme 7) would be strongly endergonic (Δ*G* = 3.36 kcal/mol, *n* = 6). Related species, such as (1) (R_f_*_n_*)_3_CCF_2_CF_2_CH_2_I, which features a more remote branch site, or (2) (R_f_*_n_*CH_2_)_3_CCH_2_I, which features additional insulating methylene groups, would be more likely to give stabile iodine(III) dichlorides. Nonetheless, these types of species have never been described in the literature. Silicon has been used as a locus for branching, as exemplified by a variety of highly fluorophilic compounds of the formula (R_f_*_n_*CH_2_CH_2_)_3_SiZ (see **XI** in Scheme 7, Z = 4-C_6_H_5_X [53–54]). However, these feature silicon–carbon and sp^3^ carbon–hydrogen bonds that may be sensitive towards Cl_2_.

The fluorous aryl iodides that are precursors to **III** and **IV** have rather modest fluorophilicities (CF_3_C_6_F_11_/toluene partition coefficients (69.5–74.7):(30.5–25.3) [16]), and simply lengthening the R_f8_ segments to R_f10_ or even longer is unlikely to achieve biases of >99:<1. The same goes for 1,2,4-(R_f8_CH_2_CH_2_)_2_C_6_H_3_I in Scheme 6. Accordingly, we suggest that branched fluorous aryl iodides of the formula (R_f_*_n_*)_3_CC_6_H_4_I (**XII**, Scheme 7) have particular promise. DFT calculations establish exergonic chlorinations, with Δ*G* values of −1.93 and −1.59 kcal/mol for *n* = 6 and 8. This implies that the corresponding iodine(III) dichlorides should have good stabilities, equal to or better than those of 1,3- and 1,4-R_f6_C_6_H_4_ICl_2_ in Scheme 5. However, this represents a currently unknown type of compound, and the synthesis is potentially challenging.

The following analysis of the crystal structure of 1,3,5-(R_f6_)_2_C_6_H_3_I is kept brief, as this compound crystallizes in the same space group and crystal system (*I*4, tetragonal) as the corresponding bromide 1,3,5-(R_f6_)_2_C_6_H_3_Br reported earlier [48]. The unit cell dimensions of the latter are virtually identical, with the cell volume ca. 1.5% lower (4851.7(4) vs 4920.1(3) Å^3^), apropos to the smaller bromine atom. The space group is both chiral and polar, and the unit cell dimensions of both compounds feature *c* values (5.5976(2)–5.5624 Å) that are much smaller than the *a* and *b* values (29.6474(9)–29.5335(13) Å). As noted earlier and illustrated in Figure 2 (bottom), the sixteen perfluorohexyl groups associated with the eight molecules in the unit cell lie roughly in the *a*/*b* plane. They largely segregate, as seen for most fluorous molecules [44,51,55–56], into fluorous domains.

**Figure 5:**
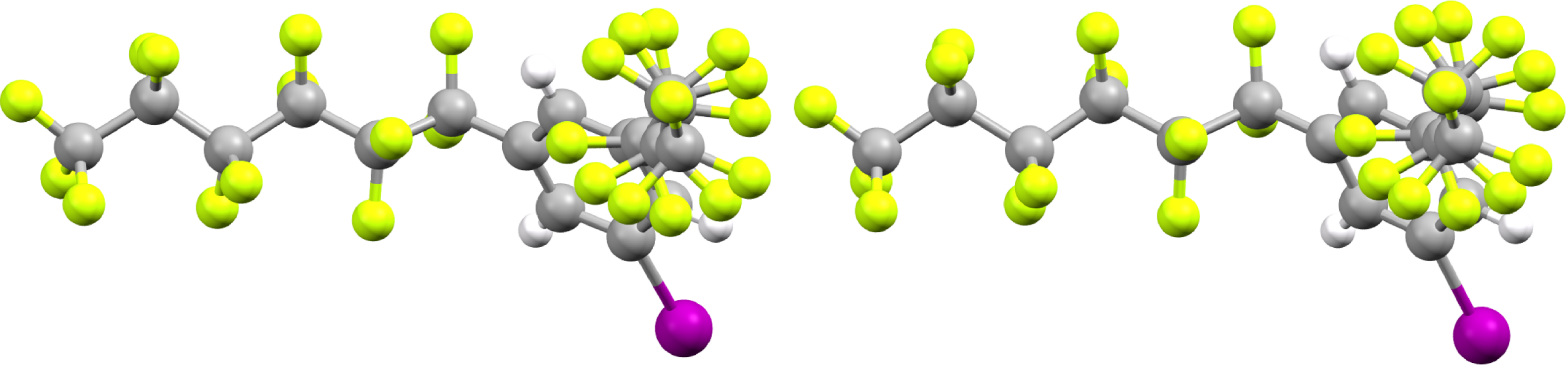
Views of the helical motif of the perfluorohexyl segments in crystalline 1,3,5-(R_f6_)_2_C_6_H_3_I (left) and the structure computed by DFT calculations (right).

The eight arene rings in the unit cell tilt distinctly out of the *a*/*b* plane (average angle 49.1°). Furthermore, the eight iodine atoms are oriented on the same "side" or *a*/*b* face of the unit cell. In the neighboring unit cell that adjoins the *a*/*b* face, the iodine atoms are found on the opposite side (*c* direction). This represents the molecular basis for the polar nature of the crystal. Also, the C–C–C–C and F–C–C–F segments in the perfluorohexyl groups do not exhibit the idealized antiperiplanar and gauche conformations associated with saturated alkanes. Rather, the torsion angles for the roughly anti linkages average 164.4(1.6)° and 166.0(3.6)°, respectively. This leads to helical motifs as shown in Figure 5, which are furthermore reproduced by the computations. The basis for this deviation, as well as a more detailed presentation of the torsional relationships, is provided elsewhere [57–59]. In a given molecule, the C_6_F_13_ groups exhibit opposite helical chiralities (see Figure S2, Supporting Information File 1), affording a *meso* stereoisomer.

Finally, attempts have been made to extend the preceding chemistry in several directions. In screening experiments, all of the fluorous iodine(III) dichlorides assayed, as well as PhICl_2_, were competent for the free radical chlorination of methane [25]. Under certain conditions, uncommon selectivities were apparent, but the fluorophilicities of the dichlorides or precursor iodides studied were insufficient for certain target recycling strategies. As discussed above, it is not clear how to meet these challenges at this time, although the couple R_f13_CH_2_ICl_2_/R_f13_CH_2_I would be one of several with promise. Regardless, the fluorous iodides reported herein have numerous other uses, some of which will be communicated in the near future [23].

## Conclusion

The preceding experimental and computational data have established a strong correlation between iodine atom Lewis basicity and the feasibility of oxidizing fluorous and non-fluorous aliphatic and aromatic iodides to the corresponding iodine(III) dichlorides. Although a few surprises are noted, these are attributed to special phenomena that can drive equilibria, such as precipitation (e.g., the conversion of **I**-Me to **II**-Me in Scheme 1), or kinetic barriers (inertness of R_fOx_CH_2_I in Scheme 3 or the precursor to **V** in Scheme 1). With the fluorous iodides, the extent of chlorination generally provides a measure of the degree to which the electron-withdrawing perfluoroalkyl or perfluoroether segments are insulated from the Lewis basic site.

## Experimental

Five syntheses that are representative of the types of transformations in this study are detailed in the main article. The remaining preparations are described in Supporting Information File 1, together with data on the solvents, starting materials, and instrumentation employed.

**R_f11_CH_2_OTf.** A Schlenk flask was flame dried, allowed to cool, charged with R_f11_CH_2_OH (5.10 g, 8.52 mmol) and anhydrous CF_3_C_6_H_5_ (50 mL) under a N_2_ flow, capped, and placed in an ice bath. Then pyridine (1.0 mL, 1.0 g, 13 mmol) and (after 30 min) Tf_2_O (3.0 mL, 5.3 g, 14 mmol) were added dropwise by syringe with stirring. The ice bath was allowed to warm to room temperature. After 16 h, H_2_O (60 mL) was added. After 30 min, the organic phase was separated and dried (MgSO_4_). The solvent was removed by rotary evaporation. The residue was dissolved in petroleum ether/ethyl acetate (4:1 v/v). The solution was filtered through a silica pad (3 × 5 cm) and the solvent was removed by rotary evaporation to give R_f11_CH_2_OTf as a white solid (3.82 g, 5.21 mmol, 61%), mp 77.2–79.9 °C (capillary). Anal. calcd for C_13_H_2_F_26_O_3_S: C, 21.33; H, 0.28; F, 67.46; S, 4.38; found: C, 21.44; H, 0.31; F, 67.21; S, 4.15; ^1^H NMR (500 MHz, acetone-*d*_6_) δ 5.55 (t, ^3^*J*_HF_ = 13 Hz, 2H, CH_2_); ^19^F{^1^H} NMR (470 MHz, acetone-*d*_6_) δ −75.5 (s, 3F, SO_2_CF_3_), −81.7 (t, ^4^*J*_FF_ = 10 Hz [60–62], 3F, CF_3_), −120.2 (m, 2F, CF_2_), −122.2 (m, 12F, 6CF_2_), −123.2 (m, 4F, 2CF_2_), −126.7 (m, 2F, CF_2_); ^13^C{^1^H} NMR (125 MHz, acetone-*d*_6_, partial) δ 70.0 (t, ^2^*J*_CF_ = 28 Hz, CH_2_); IR (powder film, cm^−1^): 2924 (w), 2855 (w), 1418 (m), 1202 (s), 1140 (s), 1103 (w), 1023 (m), 854 (m), 822 (m).

**R_f11_CH_2_I.** A round bottom flask was charged with R_f11_CH_2_OTf (3.01 g, 4.11 mmol), NaI (10.2 g, 68.0 mmol), and acetone (30 mL), and fitted with a condenser. The flask was placed in a 75 °C oil bath and the mixture was stirred. After 1 d, the bath was removed and the mixture was allowed to cool. The solvent was removed by rotary evaporation. Then Et_2_O (50 mL) and H_2_O (40 mL) were added with stirring. After 5 min, the dark brown organic phase was separated, washed with saturated aqueous Na_2_S_2_O_3_ until it became colorless, and dried (Na_2_SO_4_). The solvent was removed by rotary evaporation and the residue was dissolved in hexanes/ethyl acetate (20:1 v/v). The solution was kept at −35 °C until a precipitate formed. The solid was collected by filtration and washed with cold hexanes to give R_f11_CH_2_I as a white solid (2.00 g, 2.82 mmol, 69%), mp (capillary): 97.8–98.2 °C. Anal. calcd for C_12_H_2_F_23_I: C, 20.30; H, 0.28; F, 61.54; I, 17.87; found: C, 20.20; H, 0.16; F, 61.29; I, 17.68; ^1^H NMR (500 MHz, acetone-*d*_6_) δ 4.06 (t, ^3^*J*_HF_ = 19 Hz, 2H, CH_2_); ^19^F{^1^H} NMR (470 MHz, acetone-*d*_6_) δ −81.6 (t, ^4^*J*_FF_ = 10 Hz [60–62], 3F, CF_3_), −107.0 (m, 2F, CF_2_), −122.2 (m, 14F, 7CF_2_), −123.2 (m, 2F, CF_2_), −126.7 (m, 2F, CF_2_); ^13^C{^1^H} NMR (125 MHz, acetone-*d*_6_, partial) δ −3.8 (t, ^2^*J*_CF_ = 25 Hz, CH_2_); IR (powder film, cm^−1^): 2986 (w), 2874 (w), 1422 (w), 1373 (w), 1348 (w), 1234 (s), 1200 (s), 1140 (s), 1040 (m), 858 (m).

**R_f11_CH_2_ICl_2_.** A round bottom flask was charged with R_f11_CH_2_I (1.01 g, 1.42 mmol), C_6_F_6_ (1.4 mL), and CH_3_CN (14 mL) with stirring. Aqueous NaOCl (2.5% w/w, 21 mL) and then conc. HCl (10 mL) were slowly added. After 2 h, a pale yellow precipitate began to form. After 5 h, the mixture was filtered. The filter cake was washed with hexane (10 mL) and air dried (4–5 h) to give R_f11_CH_2_ICl_2_ as a pale yellow powder (0.90 g, 1.15 mmol, 81%), mp 122.1–125.4 °C (capillary). Anal. calcd for C_12_H_2_F_23_Cl_2_I: C, 18.46; H, 0.26; F, 55.96; Cl, 9.08; found: C, 16.76; H, 1.16; F, 50.15; Cl, 7.98 [63]; ^1^H NMR (500 MHz, acetone-*d*_6_) δ 5.44 (t, ^3^*J*_HF_ = 17 Hz, 2H, CH_2_); ^19^F{^1^H} NMR (470 MHz, acetone-*d*_6_, partial) δ −106.7 (m, 2F, CF_2_); IR (powder film, cm^−1^): 3030 (w), 2970 (w), 1392 (w), 1373 (w), 1348 (w), 1315 (w), 1202 (s), 1148 (s), 1046 (m), 860 (m).

**1,3-R_f6_C_6_H_4_I.** A Schlenk tube was charged with copper (1.26 g, 20.0 mmol) and DMSO (30 mL) and placed in a 105 °C oil bath. The mixture was sparged with N_2_ with stirring (30 min), and 1,3-diiodobenzene (6.60 g, 20.0 mmol) was added. After a second sparge, R_f6_I (4.48 g, 10.0 mmol) was added in portions over 30 min under a N_2_ flow with stirring. The tube was sealed and placed in a 110 °C oil bath. After 4 d, the mixture was cooled to room temperature and poured into H_2_O (100 mL). Then Et_2_O (100 mL) was added with stirring. After 1 h, the aqueous phase was separated and extracted with Et_2_O (5 × 50 mL). The combined organic phases were dried (Na_2_SO_4_) and the solvent was removed by rotary evaporation. The residue was dissolved in CH_3_CN (10 mL). The sample was extracted with perfluorohexane (5 × 5 mL). The fluorous layers were combined, concentrated to 2 mL, and extracted with acetone (5 × 3 mL). The solvent was removed from the extracts by oil pump vacuum to give 1,3-R_f6_C_6_H_4_I as a colorless oil (3.16 g, 6.05 mmol, 60% based upon R_f6_I). Anal. calcd for C_12_H_4_F_13_I: C; 27.61; H, 0.77; F, 47.31; found: C, 28.09; H, 0.67; F, 48.59 [63]. The solvent was removed from the concentrated perfluorohexane extract by oil pump vacuum to give 1,3-(R_f6_)_2_C_6_H_4_ as a light yellow oil (1.07 g, 1.51 mmol, 15%) [48]. The ^1^H NMR spectrum matched those in the literature [47–48]. ^1^H NMR (500 MHz, CD_2_Cl_2_) δ 8.01 (s, 1H), 7.96 (d, ^3^*J*_HH_ = 8 Hz, 1H), 7.61 (d, ^3^*J*_HH_ = 8 Hz, 1H), 7.26 (t, ^3^*J*_HH_ = 8 Hz, 1H); ^19^F{^1^H} (470 MHz, CD_2_Cl_2_) δ −82.0 (t, ^4^*J*_FF_ = 9 Hz [60–62], 3F, CF_3_), −111.7 (t, ^4^*J*_FF_ = 15 Hz [60–62], 2F, CF_2_), −122.1 (m, 2F, CF_2_), −122.3 (m, 2F, CF_2_), −123.5 (m, 2F, CF_2_), −127.0 (m, 2F, CF_2_); ^13^C{^1^H,^19^F} (125 MHz, CD_2_Cl_2_) δ 141.9, 136.4, 130.8, 126.8, 118.0 (5 × s, C_6_H_4_), 116.1, 115.8, 112.0, 111.5, 111.1, 109.3 (5 × s, 5CF_2_/CF_3_), 94.4 (s, CI).

**1,3-R_f6_C_6_H_4_ICl_2_.** A round bottom flask was charged 1,3-R_f6_C_6_H_4_I (0.523 g, 1.00 mmol), C_6_F_6_ (1 mL), and CH_3_CN (10 mL) with stirring. Aqueous NaOCl (2.5% w/w, 10 mL) followed by conc. HCl (10 mL) were slowly added. After 30 min, a pale yellow precipitate began to form. After 3 h, the mixture was filtered. The filter cake was washed with H_2_O (5 mL) and hexane (10 mL) and air dried (2 d) to give 1,3-R_f6_C_6_H_4_ICl_2_ as a pale yellow powder (0.477 g, 0.804 mmol, 80%). Anal. calcd for C_12_H_4_F_13_Cl_2_I: C, 24.31; H, 0.68; F, 41.65; found: C, 23.89; H, 0.38; F, 49.81 [63]; ^1^H NMR (500 MHz, CDCl_3_/C_6_F_6_) δ 8.54–8.52 (m, 2H), 7.94 (d, ^3^*J*_HH_ = 8 Hz, 1H), 7.79 (t, ^3^*J*_HH_ = 8 Hz, 1H); ^19^F{^1^H} NMR (470 MHz, CDCl_3_/C_6_F_6_) δ −82.3 (t, ^4^*J*_FF_ = 9 Hz [60–62], 3F, CF_3_), −112.0 (t, ^4^*J*_FF_ = 15 Hz [60–62], 2F, CF_2_), −124.4 (m, 4F, 2CF_2_), −123.7 (m, 2F, CF_2_), −127.3 (m, 2F, CF_2_).

**Partition coefficients**. The following is representative. A 20 mL vial was charged with a CF_3_C_6_F_11_ solution of R_f_*_n_*CH_2_I (*n* = 11, 15; 5.0 × 10^−2^ M, 4.0 mL) and toluene (4.0 mL), capped, and vigorously stirred. After 10 min at room temperature (24 °C), aliquots were removed from the fluorous (2.0 mL) and organic (2.0 mL) phases. The solvent was evaporated from each, and the residues were dried under vacuum. A solution of Ph_2_SiMe_2_ (internal standard; 0.0055 mL) in acetone-*d*_6_/CF_3_C_6_H_5_ (1:1 v/v; 10.0 mL) was prepared. Each residue was dissolved in 1.00 mL of this solution and ^1^H NMR spectra were recorded. The relative peak integrations gave the corresponding partition coefficients.

**Crystallography**. A solution of 1,3,5-(R_f6_)_2_C_6_H_3_I (ca. 0.05 g) in CHCl_3_/C_6_F_6_ (1.0 mL, 4:1 v/v) in an NMR tube was allowed to concentrate. After 2 d, colorless needles with well defined faces were obtained. Data were collected as outlined in Table 1. Integrated intensity information for each reflection was obtained by reduction of the data frames with the program APEX2 [64]. Data were corrected for Lorentz and polarization factors, and using SADABS [65] for absorption and crystal decay effects. The structure was solved by direct methods using SHELXTL/XS [66–67]. Non-hydrogen atoms were refined with anisotropic thermal parameters. Hydrogen atoms were placed in idealized positions and refined using a riding model. The structure was refined (weighted least squares refinement on *F*^2^) to convergence [66–68].

**Calculations.** Computations were performed with the Gaussian09 program package, employing the ultrafine grid (99,590) to enhance accuracy [69]. Geometries were optimized using density functional theory (DFT). The B3LYP [70–72] functional was employed with an all-electron 6-311+G(d,p) [73] basis set on all atoms except iodine, which was treated using an effective core potential, SDD [74]. The optimized structures were subjected to frequency calculations (using the same functional and basis set as before) to confirm that all structures were local minima and to obtain the free energies of chlorination (Figure 4 and Table S1, Supporting Information File 1).

## Supporting Information

Full details and product characterization for all the syntheses described in Schemes 2, 3, 5, and 6, information on the solvents, starting materials, and instrumentation employed, and additional spectroscopic, structural, and computational data, including a molecular structure file that can be read by the program Mercury [75] and contains the optimized geometries of all computed structures [76].

File 1 Experimental section continued.

File 2 Molecular structure file.
