# Supplementary material for: Syntheses, structures, and stabilities of aliphatic and aromatic fluorous iodine(I) and iodine(III) compounds: the role of iodine Lewis basicity
Source: Beilstein J Org Chem. 2017 Nov 23;13:2486–501. doi: 10.3762/bjoc.13.246 (PMC5704765; doi:10.3762/bjoc.13.246)
Supplement: File 1 — Experimental section continued. [file Beilstein_J_Org_Chem-13-2486-s001.pdf]

Supporting Information
for

Syntheses, structures, and stabilities of aliphatic and aromatic fluorous iodine(I) and iodine(III) compounds: the role of iodine Lewis basicity

Tathagata Mukherjee[1§], Soumik Biswas[1], Andreas Ehnbom[1], Subrata K. Ghosh[1], Ibrahim El-Zoghbi[2], Nattamai Bhuvanesh[1], Hassan S. Bazzi*[2] and John A. Gladysz*[1]

Address: [1]Department of Chemistry, Texas A&M University, P.O. Box 30012, College Station, Texas, 77842-3012, USA, and [2]Department of Chemistry, Texas A&M University at Qatar, P.O. Box 23874, Doha, Qatar

***Corresponding author**

Email: Hassan S. Bazzi - bazzi@tamu.edu; John A. Gladysz - gladysz@mail.chem.tamu.edu

[§]Present address: Dr. Tathagata Mukherjee, Intel Corporation, Hillsboro, Oregon.

Experimental section continued

General. Reactions and workups were carried out in air unless noted or Schlenk ware is specified. NMR spectra were recorded on standard FT spectrometers and referenced as follows (δ in ppm): ^{1}H, residual internal $CHCl_3$ (7.26), $CHDCl_2$ (5.32), or acetone-d_5 (2.05); $^{13}C\{^1H\}$, internal $CDCl_3$ (77.0), CD_2Cl_2 (53.8), acetone-d_6 (29.9), or C_6F_6 (140.0); $^{19}F\{^1H\}$, internal C_6-F_6 (–164.9) or 1,3,5-$(CF_3)_2C_6H_3Br$ (–63.6). IR spectra were recorded using a Shimadzu IRAffinity-1 spectrometer with a Pike MIRacle ATR system (diamond/ZnSe crystal). Microanalyses were performed by Atlantic Microlab.

Solvents were treated as follows: hexanes, Et_2O, and CH_2Cl_2, dried and degassed using a Glass Contour solvent purification system; $CF_3C_6H_5$ (Sigma Aldrich, 99%), handled under N_2; CH_3CN, distilled from CaH_2; MeOH (BDH, 99.8%); ethylacetate (BDH, ACS grade), acetone (BDH, ACS grade), petroleum ether (BDH, ACS grade), perfluorohexane (FC-72; SynQuest Laboratories), perfluoroheptane (FC-77; SynQuest Laboratories), $CF_3C_6F_{11}$ (SynQuest Laboratories), C_6F_6 (TCI, >99%), and all deuterated solvents (Cambridge Isotope Laboratories), used as received (some samples of acetone-d_6 were wet).

Chemicals were used as follows: anhydrous pyridine (Sigma Aldrich sure/sealed, 99.8%), handled under N_2; aqueous NaOCl (Ricca Chemical Company, ca. 2.5% w/w), stored in a refrigerator; Tf_2O (Sigma Aldrich, ≥99%), stored in a glove box; fluorous iodides or alcohols ($R_{fn}I$, $R_{fn}CH_2OH$, $R_{fOx}CH_2OH$; SynQuest Laboratories, 97-99%), trifluoroacetic acid (Alfa Aesar, 99%), CH_3CO_2H (BDH, ACS grade), conc. HCl (12 N, Macron/ACS grade), H_2SO_4 (36 N, BDH/ACS grade), fuming H_2SO_4 (Alfa Aesar), N-iodosuccinimide (NIS; Alfa Aesar, 97%), NaI (EMD, ACS grade), H_5IO_6 (Alfa Aesar, 99%), Ph_2SiMe_2 (>97%, TCI) and silica (SiliaFlash® F60, 230-400 mesh), used as purchased.

$R_{f11}CH_2OTf$. A Schlenk flask was flame dried, allowed to cool, charged with $R_{f11}CH_2$-OH (5.10 g, 8.52 mmol) and anhydrous $CF_3C_6H_5$ (50 mL) under a N_2 flow, capped, and placed in an ice bath. Then pyridine (1.0 mL, 1.0 g, 13 mmol) and (after 30 min) Tf_2O (3.0 mL, 5.3 g, 14 mmol) were added dropwise by syringe with stirring. The ice bath was allowed to warm to room temperature. After 16 h, H_2O (60 mL) was added. After 30 min, the organic phase was

separated and dried ($MgSO_4$). The solvent was removed by rotary evaporation. The residue was dissolved in petroleum ether/ethyl acetate (4:1 v/v). The solution was filtered through a silica pad (3 × 5 cm) and the solvent was removed by rotary evaporation to give $R_{f11}CH_2OTf$ as a white solid (3.82 g, 5.21 mmol, 61%), mp 77.2-79.9 °C (capillary). Anal. Calcd for $C_{13}H_2F_{26}O_3S$: C 21.33, H 0.28, F 67.46, S 4.38. Found C 21.44, H 0.31, F 67.21, S 4.15.

NMR (δ in ppm): ^1H (acetone-d_6, 500 MHz) 5.55 (t, $^3J_{HF}$ = 13 Hz, 2H, CH_2); $^{19}F\{^1H\}$ (acetone-d_6, 470 MHz) −75.5 (s, 3F, SO_2CF_3), −81.7 (t, $^4J_{FF}$ = 10 Hz [s1-s3], 3F, CF_3), −120.2 (m, 2F, CF_2), −122.2 (m, 12F, $6CF_2$), −123.2 (m, 4F, $2CF_2$), −126.7 (m, 2F, CF_2); $^{13}C\{^1H\}$ (acetone-d_6, 125 MHz, partial) 70.0 (t, $^2J_{CF}$ = 28 Hz, CH_2). IR (powder film, cm^{-1}): 2924 (w), 2855 (w), 1418 (m), 1202 (s), 1140 (s), 1103 (w), 1023 (m), 854 (m), 822 (m).

$R_{f13}CH_2OTf$. $R_{f13}CH_2OH$ (6.50 g, 9.29 mmol), anhydrous $CF_3C_6H_5$ (50 mL), C_6F_6 (10 mL), pyridine (1.2 mL, 1.1 g, 14 mmol), and (after 2 h) Tf_2O (3.5 mL, 5.8 g, 15 mmol) were combined in a procedure analogous to that for $R_{f11}CH_2OTf$. A similar workup (24 h prior to H_2O addition; drying with Na_2SO_4) gave $R_{f13}CH_2OTf$ as a white solid (4.64 g, 5.57 mmol, 60%) mp 105.3-106.3 °C (capillary). Anal. Calcd for $C_{15}H_2F_{30}O_3S$: C 21.65, H 0.24, S 3.85. Found C 21.71, H 0.18, S 3.71.

NMR (δ in ppm): ^1H (acetone-d_6, 500 MHz) 5.55 (t, $^3J_{HF}$ = 13 Hz, 2H, CH_2); $^{19}F\{^1H\}$ (acetone-d_6, 470 MHz) −75.5 (s, 3F, SO_2CF_3), −81.6 (t, $^4J_{FF}$ = 10 Hz [s1-s3], 3F, CF_3), −120.2 (m, 2F, CF_2), −122.2 (m, 16F, $8CF_2$), −123.3 (m, 4F, $2CF_2$), −126.8 (m, 2F, CF_2); $^{13}C\{^1H\}$ (C_6F_6, 125 MHz, partial) 68.6 (t, $^2J_{CF}$ = 27 Hz, CH_2). IR (powder film, cm^{-1}): 3000 (w), 2960 (w), 1418 (m), 1204 (s), 1142 (s), 1081 (w), 1028 (m), 893 (w), 824 (m).

$R_{f15}CH_2OTf$. A Schlenk flask was flame dried, allowed to cool, charged with $R_{f15}CH_2$-OH (10.0 g, 12.5 mmol) and anhydrous $CF_3C_6H_5$ (50 mL) under a N_2 flow, and capped. Then pyridine (1.50 mL, 1.47 g, 18.6 mmol) and (after 1 d) Tf_2O (4.50 mL, 7.61 g, 27.0 mmol) were added dropwise by syringe with stirring. After 1 d, H_2O (180 mL) was added. The mixture was filtered (filtrate = A). The filter cake was washed with acetone (10 mL) and this filtrate was discarded. The remaining filter cake was transferred to a round bottom flask and taken up in CF_3C_6-

H_5 (75 mL) with stirring. After 30 min, this mixture was filtered. This filtrate was combined with the original filtrate (A) and dried (Na_2SO_4). The solvent was removed by rotary evaporation, and hexanes/ethyl acetate (4:1 v/v, 500 mL) was added. The flask was placed in a 50 °C oil bath. The mixture was stirred until the solid completely dissolved. The solution was kept at –35 °C until a precipitate formed. After 8-9 h, the solid was collected by filtration and washed with cold hexanes to give $R_{f15}CH_2OTf$ as a white solid (2.61 g, 2.80 mmol, 22%), mp 127.4-128.2 °C (capillary). Anal. Calcd for $C_{17}H_2F_{34}O_3S$: C 21.90, H 0.22, F 69.29, S 3.44. Found C 21.01, H 0.13, F 66.62, S 3.52 [s4].

NMR (δ in ppm): ^1H (acetone-d_6, 500 MHz) 5.48 (t, $^3J_{HF}$ = 13 Hz, 2H, CH_2); $^{19}F\{^1H\}$ (acetone-d_6, 470 MHz) −75.5 (s, 3F, SO_2CF_3), −81.6 (t, $^4J_{FF}$ = 10 Hz [s1-s3], 3F, CF_3), −120.2 (m, 2F, CF_2), −122.1 (m, 20F, $10CF_2$), −123.2 (m, 4F, $2CF_2$), −126.7 (m, 2F, CF_2); $^{13}C\{^1H\}$ (acetone-d_6/C_6F_6, 125 MHz, partial) 68.6 (t, $^2J_{CF}$ = 28 Hz, CH_2). IR (powder film, cm^{-1}): 2922 (w), 2855 (w), 1418 (m), 1204 (s), 1144 (s), 1099 (w), 1028 (m), 872 (w), 825 (m).

$R_{f11}CH_2I$. A round bottom flask was charged with $R_{f11}CH_2OTf$ (3.01 g, 4.11 mmol), NaI (10.2 g, 68.0 mmol), and acetone (30 mL), and fitted with a condenser. The flask was placed in a 75 °C oil bath and the mixture was stirred. After 1 d, the bath was removed and the mixture was allowed to cool. The solvent was removed by rotary evaporation. Then Et_2O (50 mL) and H_2O (40 mL) were added with stirring. After 5 min, the dark brown organic phase was separated, washed with saturated aqueous $Na_2S_2O_3$ until it became colorless, and dried (Na_2SO_4). The solvent was removed by rotary evaporation and the residue was dissolved in hexanes/ethyl acetate (20:1 v/v). The solution was kept at –35 °C until a precipitate formed. The solid was collected by filtration and washed with cold hexanes to give $R_{f11}CH_2I$ as a white solid (2.00 g, 2.82 mmol, 69%), mp (capillary): 97.8-98.2 °C. Anal. Calcd for $C_{12}H_2F_{23}I$: C 20.30, H 0.28, F 61.54, I 17.87. Found: C 20.20, H 0.16, F 61.29, I 17.68.

NMR (δ in ppm, acetone-d_6): ^1H (500 MHz) 4.06 (t, $^3J_{HF}$ = 19 Hz, 2H, CH_2); $^{19}F\{^1H\}$ (470 MHz) −81.6 (t, $^4J_{FF}$ = 10 Hz [s1-s3], 3F, CF_3), −107.0 (m, 2F, CF_2), −122.2 (m, 14F, $7CF_2$), −123.2 (m, 2F, CF_2), −126.7 (m, 2F, CF_2); $^{13}C\{^1H\}$ (125 MHz. partial) −3.8 (t, $^2J_{CF}$ =

25 Hz, CH_2). IR (powder film, cm^{-1}): 2986 (w), 2874 (w), 1422 (w), 1373 (w), 1348 (w), 1234 (s), 1200 (s), 1140 (s), 1040 (m), 858 (m).

$R_{f13}CH_2I$. A sample of $R_{f13}CH_2OTf$ (0.335 g, 0.403 mmol), NaI (3.59 g, 23.9 mmol), and acetone (8 mL) were combined in a procedure analogous to that for $R_{f11}CH_2I$. A similar workup (Et_2O/30 mL and H_2O/20 mL; after the addition of hexanes/ethyl acetate, the flask was placed in a 50 °C oil bath and the mixture stirred until the solid completely dissolved) gave R_{f13}-CH_2I as a white solid (0.190 g, 0.235 mmol, 58%), mp 124.4-124.9 °C (capillary). Anal. Calcd for $C_{14}H_2F_{27}I$: C 20.76, H 0.25, F 63.33, I 15.67. Found C 20.81, H 0.20, F 63.33, I 15.48.

NMR (δ in ppm): ^1H (acetone-d_6, 500 MHz) 4.06 (t, $^3J_{HF}$ = 19 Hz, 2H, CH_2); $^{19}F\{^1H\}$ (acetone-d_6, 470 MHz) −81.7 (t, $^4J_{FF}$ = 10 Hz [s1-s3], 3F, CF_3), −107.0 (m, 2F, CF_2), −122.2 (m, 18F, 9CF_2), −123.2 (m, 2F, CF_2), −126.7 (m, 2F, CF_2); $^{13}C\{^1H\}$ (C_6F_6, 125 MHz, partial) −7.0 (t, $^2J_{CF}$ = 26 Hz, CH_2). IR (powder film, cm^{-1}): 1422 (w), 1375 (w), 1354 (w), 1201 (s), 1144 (s), 1045 (m), 812 (m).

$R_{f15}CH_2I$. A sample of $R_{f15}CH_2OTf$ (0.282 g, 0.303 mmol), NaI (1.45 g, 9.67 mmol), and acetone (5 mL) were combined in a procedure analogous to that for $R_{f11}CH_2I$. A similar workup (Et_2O/20 mL and H_2O/15 mL) gave $R_{f15}CH_2I$ as a white solid (0.166 g, 0.182 mmol, 60%), mp 146.0-146.5 °C (capillary). Anal. Calcd for $C_{16}H_2F_{31}I$: C 21.12, H 0.22, F 64.72. Found: C 21.42, H 0.23, F 64.44.

NMR (δ in ppm): ^1H (acetone-d_6, 500 MHz) 4.06 (t, $^3J_{HF}$ = 18 Hz, 2H, CH_2); $^{19}F\{^1H\}$ (acetone-d_6, 470 MHz) −81.6 (t, $^4J_{FF}$ = 10 Hz [s1-s3], 3F, CF_3), −107.0 (m, 2F, CF_2), −122.1 (m, 22F, 11CF_2), −123.2 (m, 2F, CF_2), −126.7 (m, 2F, CF_2); $^{13}C\{^1H\}$ (C_6F_6, 125 MHz, partial) −7.0 (t, $^2J_{CF}$ = 26 Hz, CH_2). IR (powder film, cm^{-1}): 2922 (w), 2853 (w), 1420 (w), 1377 (w), 1335 (w), 1204 (s), 1148 (s), 1049 (m), 870 (w).

$R_{f11}CH_2ICl_2$. A round bottom flask was charged with $R_{f11}CH_2I$ (1.01 g, 1.42 mmol), C_6F_6 (1.4 mL), and CH_3CN (14 mL) with stirring. Aqueous NaOCl (2.5% w/w, 21 mL) and then conc. HCl (10 mL) were slowly added. After 2 h, a pale yellow precipitate began to form. After 5 h, the mixture was filtered. The filter cake was washed with hexane (10 mL) and air dried (4-5 h)

to give $R_{f11}CH_2ICl_2$ as pale yellow powder (0.90 g, 1.15 mmol, 81%), mp 122.1-125.4 °C (capillary). Anal. Calcd for $C_{12}H_2F_{23}Cl_2I$: C 18.46, H 0.26, F 55.96, Cl 9.08. Found C 16.76, H 1.16, F 50.15, Cl 7.98 [s4].

NMR (δ in ppm, acetone-d_6): ^{1}H (500 MHz) 5.44 (t, $^3J_{HF}$ = 17 Hz, 2H, CH_2); ^{19}F{^{1}H} (470 MHz, partial) −106.7 (m, 2F, CF_2). IR (powder film, cm^{-1}): 3030 (w), 2970 (w), 1392 (w), 1373 (w), 1348 (w), 1315 (w), 1202 (s), 1148 (s), 1046 (m), 860 (m).

$R_{f13}CH_2ICl_2$. A sample of $R_{f13}CH_2I$ (0.147 g, 0.178 mmol), C_6F_6 (5 mL), CH_3CN (3.5 mL), aqueous NaOCl (2.5% w/w, 3 mL), and conc. HCl (2 mL) were combined in a procedure analogous to that for $R_{f11}CH_2ICl_2$. An identical workup gave $R_{f13}CH_2ICl_2$ as a pale yellow powder (0.052 g, 0.059 mmol, 33%), mp 164.1-166.2 °C (capillary).

NMR (δ in ppm, acetone-d_6): ^{1}H (500 MHz) 5.43 (t, $^3J_{HF}$ = 17 Hz, 2H, CH_2); ^{19}F{^{1}H} (470 MHz, partial) −106.7 (m, 2F, CF_2). IR (powder film, cm^{-1}): 3038 (w), 2970 (w), 1391 (w), 1377 (w), 1356 (w), 1325 (w), 1202 (s), 1146 (s), 1057 (m), 810 (m).

$R_{fO2}CH_2OTf$. A Schlenk flask was flame dried, allowed to cool, charged with $R_{fO2}CH_2$-OH (4.66 g, 7.18 mmol) and anhydrous $CF_3C_6H_5$ (40 mL) under a N_2 flow, capped, and placed in an ice bath. Then pyridine (0.90 mL, 0.88 g, 11.1 mmol) and (after 30 min) Tf_2O (2.60 mL, 4.46 g, 15.8 mmol) were added dropwise by syringe with stirring. The ice bath was allowed to warm to room temperature. After 15 h, H_2O (50 mL) was added. After 30 min, the organic phase was separated and the organic phase was dried (Na_2SO_4). The solvent was removed by rotary evaporation. The residue was dissolved in hexane/ethyl acetate (4:1 v/v). The solution was filtered through a silica pad (3 × 5 cm). The solvent was removed by rotary evaporation to give R_{fO2}-CH_2OTf as a colorless oil (5.18 g, 6.64 mmol, 92%). Anal. Calcd for $C_{13}H_2F_{26}O_6S$: C 20.01, H 0.26, F 63.31, S 4.11. Found C 20.28, H 0.17, F 63.36, S 3.95.

NMR (δ in ppm, acetone-d_6): ^{1}H (500 MHz) 5.57 (d, $^3J_{HF}$ = 11 Hz, 2H, CH_2); ^{19}F {^{1}H} (470 MHz) −75.6 to −75.7 (m [s5], 3F, SO_2CF_3), −80.4 to −83.2 (m, 18F), −130.5 (m, 2F), −135.9 to −136.5 (m, 1F), −145.3 to −145.9 (m, 2F); ^{13}C{^{1}H} (125 MHz, partial) 69.8 (d, $^2J_{CF}$ = 33 Hz, CH_2). IR (oil film, cm^{-1}): 2990 (w), 1437 (m), 1306 (m), 1229 (s), 1200 (s), 1140 (s), 991

(s), 745 (m).

$R_{fO3}CH_2OTf$. A sample of $R_{fO3}CH_2OH$ (4.89 g, 5.97 mmol), anhydrous $CF_3C_6H_5$ (30 mL), pyridine (0.75 mL, 0.73 g, 9.17 mmol), and Tf_2O (2.20 mL, 3.71 g, 13.1 mmol) were combined in a procedure analogous to that for $R_{fO2}CH_2OTf$. An identical workup gave $R_{fO3}CH_2OTf$ as a colorless oil (5.24 g, 5.54 mmol, 93%). Anal. Calcd for $C_{16}H_2F_{32}O_7S$: C 20.31, H 0.21, F 64.25, S 3.39. Found: C 20.90, H 0.15, F 65.82, S 2.46 [s4].

NMR (δ in ppm, acetone-d_6): ^1H (500 MHz) 5.57 (d, $^3J_{HF}$ = 11 Hz, 2H, CH_2); $^{19}F\{^1H\}$ (470 MHz) −75.6 to −75.7 (m [s5], 3F, SO_2CF_3), −80.3 to −83.1 (m, 22F), −130.5 (m, 2F), −135.9 to −136.6 (m, 1F), −145.0 to −145.9 (m, 4F); $^{13}C\{^1H\}$ (125 MHz, partial) 69.8 (d, $^2J_{CF}$ = 32 Hz, CH_2). IR (oil film, cm^{-1}): 2990 (w), 1802 (w), 1439 (m), 1302 (m), 1306 (s), 1198 (s), 1126 (s), 982 (s), 746 (m).

$R_{fO4}CH_2OTf$. A sample of $R_{fO4}CH_2OH$ (4.92 g, 5.02 mmol), anhydrous $CF_3C_6H_5$ (50 mL), pyridine (0.620 mL, 0.609 g, 7.67 mmol) and Tf_2O (1.89 mL, 3.17 g, 11.2 mmol) were combined in a procedures analogous to that for $R_{fO2}CH_2OTf$. An identical workup gave R_{fO4}-CH_2OTf as a colorless oil (4.80 g, 4.32 mmol, 84%). Anal. Calcd for $C_{19}H_2F_{38}O_8S$: C 20.52, H 0.18, F 64.91, S 2.88. Found C 20.68, H 0.15, F 64.98, S 3.01.

NMR (δ in ppm, acetone-d_6): ^1H (500 MHz) 5.58 (d, $^3J_{HF}$ = 12 Hz, 2H, CH_2); $^{19}F\{^1H\}$ (470 MHz) −75.6 to −75.7 (m [s5], 3F, SO_2CF_3), −80.3 to −83.1 (m, 28F), −130.4 (m, 2F), −136.3 (m, 1F), −145.0 to −145.8 (m, 4F); $^{13}C\{^1H\}$ (125 MHz, partial) 69.8 (d, $^2J_{CF}$ = 28 Hz, CH_2). IR (oil film, cm^{-1}): 2992 (w), 1802 (w), 1437 (m), 1306 (m), 1227 (s), 1198 (s), 1126 (s), 982 (s), 746 (m).

$R_{fO5}CH_2OTf$. A sample of $R_{fO5}CH_2OH$ (1.90 g, 1.66 mmol), anhydrous $CF_3C_6H_5$ (10 mL), pyridine (0.21 mL, 0.21 g, 2.6 mmol) and Tf_2O (0.62 mL, 1.03 g, 3.66 mmol) were combined in a procedure analogous to that for $R_{fO2}CH_2OTf$. As identical workup gave $R_{fO5}CH_2OTf$ as a colorless oil (1.91 g, 1.49 mmol, 90%). Anal. Calcd for $C_{22}H_2F_{44}O_9S$: C 20.67, H 0.16, F 65.40, S 2.51. Found C 20.86, H 0.14, F 65.13, S 2.34.

NMR (δ in ppm, acetone-d_6): ^1H (500 MHz) 5.57 (d, $^3J_{HF}$ = 12 Hz, 2H, CH_2); $^{19}F\{^1H\}$

(470 MHz) −75.6 to −75.7 (m [s5], 3F, SO_2CF_3), −80.3 to −83.1 (m, 32F), −130.4 (m, 2F), −136.0 to −136.5 (m, 1F), −145.0 to −145.9 (m, 4F); $^{13}C\{^1H\}$ (125 MHz, partial) 69.8 (d, $^2J_{CF}$ = 30 Hz, CH_2). IR (oil film, cm^{-1}): 2916 (w), 1439 (m), 1304 (m), 1227 (s), 1200 (s), 1125 (s), 982 (s), 745 (m).

$R_{fO2}CH_2I$. A round bottom flask was charged with $R_{fO2}CH_2OTf$ (4.56 g, 5.84 mmol), NaI (14.02 g, 93.52 mmol), and acetone (40 mL), and fitted with a condenser. The flask was placed in a 70 °C oil bath and the mixture was stirred. After 1 h, the bath was removed. After 9 h, the solvent was removed by rotary evaporation. Then Et_2O (70 mL) was added with stirring. After 15 min, the mixture was filtered. The filtrate was washed with H_2O (50 mL) and saturated aqueous $Na_2S_2O_3$ until it became colorless, and dried (Na_2SO_4). The solvent was removed by rotary evaporation and the residue was dissolved in pentane/ethyl acetate (20:1 v/v). The solution was filtered through a silica pad (3 × 8 cm). The solvent was removed by rotary evaporation to give $R_{fO2}CH_2I$ as a colorless liquid (3.66 g, 4.73. mmol, 81%).

NMR (δ in ppm, acetone-d_6): ^1H (500 MHz) 3.93-4.17 (m, 2H, CH_2); $^{13}C\{^1H\}$ (125 MHz, partial) −4.2 (m, CH_2).

$R_{fO4}CH_2I$. A round bottom flask was charged with $R_{fO4}CH_2OTf$ (3.00 g, 2.69 mmol), NaI (6.20 g, 41.4 mmol), and acetone (10 mL), and fitted with a condenser. The flask was placed in a 75 °C oil bath and the mixture was stirred. After 1 h, the bath was removed and the mixture was allowed to cool. The solvent was removed by rotary evaporation. Then Et_2O (20 mL) and H_2O (15 mL) were added with stirring. After 5 min, the dark brown organic phase was separated, washed with saturated aqueous $Na_2S_2O_3$ until it became colorless, and dried (Na_2SO_4). The solvent was removed by rotary evaporation and the residue was dissolved in hexanes/ethyl acetate (20:1 v/v). The solution was filtered through a silica pad (3 × 5 cm). The solvent was removed by rotary evaporation to give $R_{fO4}CH_2I$ as a colorless liquid (2.67 g, 2.45 mmol, 91%). Anal. Calcd for $C_{18}H_2F_{35}IO_5$: C 19.83, H 0.18, F 61.00, I 11.64. Found C 20.10, H 0.18, F 61.25, I 11.36.

NMR (δ in ppm, acetone-d_6): ^1H (500 MHz) 4.00-4.15 (m, 2H, CH_2); $^{19}F\{^1H\}$ (470 MHz) −78.7 to −85.1 (m, 28F), −122.9 to −123.5 (m, 1F), −130.4 (s, 2F), −144.8 to −145.8 (m,

4F); $^{13}C\{^1H\}$ (125 MHz, partial) −4.2 (apparent t, $^2J_{CF}$ = 22 Hz, CH_2 [s5,s6]). IR (oil film, cm^{-1}): 2976 (w), 1802 (w), 1304 (s), 1229 (s), 1200 (s), 1178 (s), 1142 (s), 1123 (s), 982 (s), 745 (m).

$R_{fO5}CH_2I$. Acetone (10 mL), $R_{fO5}CH_2OTf$ (1.72 g, 1.35 mmol), and NaI (3.22 g, 21.5 mmol) were combined in a procedure analogous to that for $R_{fO2}CH_2I$. A similar reaction and workup (3 × 5 cm silica pad) gave $R_{fO5}CH_2I$ as a colorless liquid (1.44 g, 1.13 mmol, 84%).

NMR (δ in ppm, 4:1 v/v acetone-d_6/C_6F_6): ^1H (500 MHz) 3.84-4.19 (m, 2H, CH_2); $^{13}C\{^1H\}$ (125 MHz, partial) −4.3 (m, CH_2).

1,3-$R_{f6}C_6H_4I$. A Schlenk tube was charged with copper (1.26 g, 20.0 mmol) and DMSO (30 mL) and placed in a 105 °C oil bath. The mixture was sparged with N_2 with stirring (30 min), and 1,3-diiodobenzene (6.60 g, 20.0 mmol) was added. After a second sparge, $R_{f6}I$ (4.48 g, 10.0 mmol) was added in portions over 30 min under a N_2 flow with stirring. The tube was sealed and placed in a 110 °C oil bath. After 4 d, the mixture was cooled to room temperature and poured into H_2O (100 mL). Then Et_2O (100 mL) was added with stirring. After 1 h, the aqueous phase was separated and extracted with Et_2O (5 × 50 mL). The combined organic phases were dried (Na_2SO_4) and the solvent was removed by rotary evaporation. The residue was dissolved in CH_3CN (10 mL). The sample was extracted with perfluorohexane (5 × 5 mL). The fluorous layers were combined, concentrated to 2 mL, and extracted with acetone (5 × 3 mL). The solvent was removed from the extracts by oil pump vacuum to give 1,3-$R_{f6}C_6H_4I$ as a colorless oil (3.16 g, 6.05 mmol, 60% based upon $R_{f6}I$). Anal. Calcd for $C_{12}H_4F_{13}I$: C 27.61, H 0.77, F 47.31. Found C 28.09, H 0.67, F 48.59 [s4]. The solvent was removed from the concentrated perfluorohexane extract by oil pump vacuum to give 1,3-$(R_{f6})_2C_6H_4$ as a light yellow oil (1.07 g, 1.51 mmol, 15%) [s7]. The ^1H NMR spectrum matched those in the literature [s7,s8].

NMR (δ in ppm, CD_2Cl_2): ^1H (500 MHz) 8.01 (s, 1H), 7.96 (d, $^3J_{HH}$ = 8 Hz, 1H), 7.61 (d, $^3J_{HH}$ = 8 Hz, 1H), 7.26 (t, $^3J_{HH}$ = 8 Hz, 1H); $^{19}F\{^1H\}$ (470 MHz) –82.0 (t, $^4J_{FF}$ = 9 Hz [s1-s3], 3F, CF_3), –111.7 (t, $^4J_{FF}$ = 15 Hz [s1-s3], 2F, CF_2), –122.1 (m, 2F, CF_2), –122.3 (m, 2F, CF_2), –123.5 (m, 2F, CF_2), –127.0 (m, 2F, CF_2); $^{13}C\{^1H,^{19}F\}$ (125 MHz) 141.9, 136.4, 130.8,

126.8, 118.0 (5 × s, C_6H_4), 116.1, 115.8, 112.0, 111.5, 111.1, 109.3 (5 × s, $5CF_2/CF_3$), 94.4 (s, CI).

1,3-$R_{f6}C_6H_4ICl_2$. A round bottom flask was charged 1,3-$R_{f6}C_6H_4I$ (0.523 g, 1.00 mmol), C_6F_6 (1 mL), and CH_3CN (10 mL) with stirring. Aqueous NaOCl (2.5% w/w, 10 mL) followed by conc. HCl (10 mL) were slowly added. After 30 min, a pale yellow precipitate began to form. After 3 h, the mixture was filtered. The filter cake was washed with H_2O (5 mL) and hexane (10 mL) and air dried (2 d) to give 1,3-$R_{f6}C_6H_4ICl_2$ as a pale yellow powder (0.477 g, 0.804 mmol, 80%). Anal. Calcd for $C_{12}H_4F_{13}Cl_2I$: C 24.31, H 0.68, F 41.65. Found C 23.89, H 0.38, F 49.81 [s4].

NMR (δ in ppm, $CDCl_3/C_6F_6$): ^1H (500 MHz) 8.54-8.52 (m, 2H), 7.94 (d, $^3J_{HH}$ = 8 Hz, 1H), 7.79 (t, $^3J_{HH}$ = 8 Hz, 1H); $^{19}F\{^1H\}$ (470 MHz) –82.3 (t, $^4J_{FF}$ = 9 Hz [s1-s3], 3F, CF_3), –112.0 (t, $^4J_{FF}$ = 15 Hz [s1-s3], 2F, CF_2), –124.4 (m, 4F, $2CF_2$), –123.7 (m, 2F, CF_2), –127.3 (m, 2F, CF_2).

1,4-$R_{f6}C_6H_4I$. Copper (1.26 g, 20.0 mmol), DMSO (30 mL), 1,4-diiodobenzene (6.60 g, 20.0 mmol), and $R_{f6}I$ (4.48 g, 10.0 mmol) were combined in a procedure analogous to that for 1,3-$R_{f6}C_6H_4I$. An identical workup gave 1,4-$R_{f6}C_6H_4I$ as a colorless oil (2.58 g, 4.98 mmol, 50% based upon $R_{f6}I$). Anal. Calcd for $C_{12}H_4F_{13}I$: C 27.61, H 0.77, F 47.31. Found C 27.88, H 0.65, F 47.05. The solvent was removed from the concentrated perfluorohexane extract by oil pump vacuum to give 1,4-$(R_{f6})_2C_6H_4$ as a light yellow oil (1.42 g, 1.99 mmol, 20%). Anal. Calcd for $C_{18}H_4F_{26}$: C 30.27, H 0.56, F 69.16. Found C 30.07, H 0.42, F 68.90.

NMR (δ in ppm, CD_2Cl_2): ^1H (500 MHz) 7.90 (d, $^3J_{HH}$ = 9 Hz, 2H), 7.33 (d, $^3J_{HH}$ = 9 Hz, 2H); $^{19}F\{^1H\}$ (470 MHz) –81.8 (t, $^4J_{FF}$ = 10 Hz [s1-s3], 3F, CF_3), –111.8 (t, $^4J_{FF}$ = 15 Hz [s1-s3], 2F, CF_2), –122.0 (m, 2F, CF_2), –122.5 (m, 2F, CF_2), –123.4 (m, 2F, CF_2), –126.9 (m, 2F, CF_2); $^{13}C\{^1H,^{19}F\}$ (125 MHz) 137.4, 127.8, 127.6 (3 × s, C_6H_4), 116.6, 115.2, 110.6, 110.1, 109.7, 107.9 (5 × s, $5CF_2/CF_3$), 98.4 (s, CI).

1,4-$R_{f6}C_6H_4ICl_2$. The iodide 1,4-$R_{f6}C_6H_4I$ (0.527 g, 1.01 mmol), C_6F_6 (1 mL), CH_3CN (10 mL), NaOCl (2.5% w/w, 10 mL), and HCl (10 mL) were combined in a procedure analogous

to that for 1,3-$R_{f6}C_6H_4ICl_2$. An identical workup gave 1,4-$R_{f6}C_6H_4ICl_2$ as a pale yellow powder (0.518 g, 0.873 mmol, 86%).

NMR (δ in ppm, $CDCl_3/C_6F_6$): ^1H (500 MHz) 8.46 (d, $^3J_{HH}$ = 8 Hz, 2H), 7.81 (d, $^3J_{HH}$ = 8 Hz, 2H); $^{19}F\{^1H\}$ (470 MHz) –82.3 (t, $^4J_{FF}$ = 10 Hz [s1-s3], 3F, CF_3), –112.3 (t, $^4J_{FF}$ = 14 Hz [s1-s3], 2F, CF_2), –122.3 (m, 4F, $2CF_2$), –123.7 (m, 2F, CF_2), –127.2 (m, 2F, CF_2).

1,3-$R_{f10}C_6H_4I$. Copper (1.26 g, 20.0 mmol), DMSO (30 mL), 1,3-diiodobenzene (6.40 g, 20.0 mmol), and $R_{f10}I$ (6.48 g, 10.0 mmol) were combined in a procedure similar to that for 1,3-$R_{f6}C_6H_4I$ (130 °C oil bath). The workup was identical until the organic phases were dried (Na_2SO_4). The solvent was removed by rotary evaporation, and the residue was dissolved in MeOH (40 mL) in a round bottom flask, which was capped. After 4 d, the precipitate was collected by filtration, washed with cold MeOH (1 mL), and dried by oil pump vacuum to give 1,3-R_{f10}-C_6H_4I as a white solid (3.62 g, 5.00 mmol, 50%). Anal. Calcd for $C_{16}H_4F_{21}I$: C 26.61, H 0.56, F 55.25. Found C 26.62, H 0.39, F 54.98.

NMR (δ in ppm, $CDCl_3$): ^1H (500 MHz) 7.93-7.92 (m, 2H), 7.56 (d, $^3J_{HH}$ = 8 Hz, 1H), 7.25 (t, $^3J_{HH}$ = 8 Hz, 1H); $^{19}F\{^1H\}$ (470 MHz) –80.7 (t, $^4J_{FF}$ = 10 Hz [s1-s3], 3F, CF_3), –110.9 (t, $^4J_{FF}$ = 15 Hz [s1-s3], 2F, CF_2), –121.2 (m, 2F, CF_2), –121.6 to –121.9 (m, 10F, $5CF_2$), –122.7 (m, 2F, CF_2), –126.1 (m, 2F, CF_2); $^{13}C\{^1H,^{19}F\}$ (125 MHz, partial) 141.1, 135.6, 130.2, 128.5, 126.0 (5 × s, C_6H_4), 93.7 (s, CI).

1,3-$R_{f10}C_6H_4ICl_2$. The iodide 1,3-$R_{f10}C_6H_4I$ (0.739 g, 1.02 mmol), C_6F_6 (1 mL), CH_3-CN (10 mL), NaOCl (2.5% w/w, 10 mL), and conc. HCl (10 mL) were combined in a procedure analogous to that for 1,3-$R_{f6}C_6H_4ICl_2$. An identical workup gave 1,3-$R_{f10}C_6H_4ICl_2$ as a pale yellow solid (0.724 g, 0.912 mmol, 89%). Anal. Calcd for $C_{16}H_4F_{21}ICl_2$: C 24.23, H 0.51, F 50.31. Found C 24.13, H 0.34, F 50.06. Further characterization or analysis was complicated by the extremely low solubility of this compound in common organic and fluorous solvents.

1,3,5-$(R_{f6})_2C_6H_3I$. A round bottom flask was charged with 1,3-$(R_{f6})_2C_6H_4$ (0.555 g, 0.775 mmol) [s7], fuming H_2SO_4 (0.7 mL), and trifluoroacetic acid (1.7 mL) with stirring. Then NIS (0.112 g, 0.500 mmol) was added. Identical quantities of NIS were added after 2 h and 4 h.

After 48 h, ice (30 g) was slowly added, followed by CH_2Cl_2 (100 mL). The biphasic mixture was stirred, and NaOH was added until the pH of the aqueous phase reached 7. The aqueous phase was separated and extracted with CH_2Cl_2 (3 × 50 mL). The combined organic phases were washed with 0.1 M aqueous $Na_2S_2O_3$ and dried ($MgSO_4$). The solvent was removed by rotary evaporation to give 1,3,5-$(R_{f6})_2C_6H_3I$ as a white solid (0.501 g, 0.596 mmol, 77%). Anal. Calcd for $C_{18}H_3F_{26}I$: C 25.74, H 0.36, F 58.80. Found C 25.66, H 0.24, F 58.53.

NMR (δ in ppm): ^1H (500 MHz, $CDCl_3$ or CD_2Cl_2/C_6F_6) 8.15 (s, 2H), 7.77 (s, 1H); $^{19}F\{^1H\}$ (470 MHz, CD_2Cl_2/C_6F_6) –82.7 (t, $^4J_{FF}$ = 10.5 Hz [s1-s3], 3F, CF_3), –113.2 (t, $^4J_{FF}$ = 14.5 Hz [s1-s3], 2F, CF_2), –123.4 (m, 2F, CF_2), –123.6 (m, 2F, CF_2), –124.8 (m, 2F, CF_2), –128.1 (m, 2F, CF_2); $^{13}C\{^1H\}$ (125 MHz, CD_2Cl_2/C_6F_6, partial) 132.2 (t, $^2J_{CF}$ = 25.1 Hz, CH**C**CF_2), 130.1 (s, **C**CI), 125.6 (t, $^3J_{CF}$ = 7.0 Hz, CF_2**C**CHCCF_2), 94.2 (s, CI).

1,3,5-$(R_{f6})_2C_6H_3ICl_2$. A Schlenk tube was charged with 1,3,5-$(R_{f6})_2C_6H_3I$ (1.69 g, 2.00 mmol) and $CF_3C_6H_5$ (30 mL) and cooled to –30 °C. The solution was saturated with Cl_2 gas. After 24 h, two aliquots (1 mL each) were removed and analyzed by ^1H NMR. The spectra showed 77:23 $ArICl_2$/ArI mixtures. The solvent was removed from one aliquot by rotary evaporation to give a 75:25 $ArICl_2$/ArI mixture as a pale white solid (Figure 1-b). This solid was dissolved in $CF_3C_6H_5$ (2 mL) and the sample was kept at –35 °C. After 7 d, the solvent was removed by rotary evaporation to give a 65:35 $ArICl_2$/ArI mixture as a pale white solid (Figure 1-d). The solvent was removed from the second aliquot at –40 °C by oil pump vacuum to give a 35:65 $ArICl_2$/ArI mixture as a pale white solid (Figure 1-c).

NMR (δ in ppm, $CDCl_3$): ^1H (500 MHz) 8.69 (s, 2H), 7.98 (s, 1H).

1,2,4-$(R_{f8}CH_2CH_2)_2C_6H_3I$ [s9]**.** A Schlenk tube was charged with 1,3-$(R_{f8}CH_2CH_2)_2$-C_6H_4 (0.600 g, 0.618 mmol) [s10], I_2 (0.627 g, 2.47 mmol), H_5IO_6 (0.283 g, 1.24 mmol), CH_3-CO_2H (10 mL), H_2SO_4 (0.30 mL) and H_2O (2.0 mL). The tube was placed in a 100 °C oil bath and the mixture was stirred overnight. Another charge of H_5IO_6 (0.283 g, 1.24 mmol) was added. After 12 h, the mixture was allowed to cool to room temperature, and CH_2Cl_2 (15 mL) was added. After 5 min, the purple organic phase was separated. A saturated aqueous $Na_2S_2O_3$ solu-

tion was added dropwise with vigorous stirring until the color was discharged. The aqueous phase was extracted with CH_2Cl_2 (2 × 15 mL). The combined organic phases were dried (Mg-SO_4). The solvent was removed by rotary evaporation to give 1,2,4-($R_{f8}CH_2CH_2)_2C_6H_3I$ as a white solid (0.630 g, 0.574 mmol, 93%), mp 37-39 °C (capillary). Anal. Calcd for $C_{26}H_{11}F_{34}I$: C 28.49, H 1.01, F 58.92. Found: C 28.47, H 1.13, F 58.64.

NMR (δ in ppm, $CDCl_3$): ^{1}H (400 MHz) 7.78 (d, $^3J_{HH}$ = 8 Hz, 1H, CH_{Ar}), 7.12 (s, 1H, CH_{Ar}), 6.82 (d, $^3J_{HH}$ = 8 Hz, 1H, CH_{Ar}), 2.85-3.04 (m, 4H, $2CH_2CF_2$), 2.35-2.38 (m, 4H, $2C\mathbf{H}_2CH_2CF_2$); $^{13}C\{^1H\}$ (100 MHz) 140.29, 140.24, 140.17, 129.7, 128.7 (5 × s, C_6H_3), 117.9, 116.2 (2 × t, $^2J_{CF}$ = 22 Hz, $2CF_2\mathbf{C}F_3$ [s11]), 112.8, 111.1, 110.8, 109.1, 108.3 (5 overlapping m, $7\mathbf{C}F_2$), 97.5 (s, CI), 32.7, 31.6 (2 × t, $^2J_{CF}$ = 22 Hz, $2\mathbf{C}H_2CF_2$), 26.00, 25.99 (2 × s, $2\mathbf{C}H_2CH_2CF_2$); $^{19}F\{^1H\}$ (376 MHz) –80.9 (t, $^4J_{FF}$ = 10 Hz [s1-s3], 6F, $2CF_3$), –114.2 (m, 4F, $2CF_2$), –121.5 (m, 8F, $4CF_2$), –21.7 (m, 4F, $2CF_2$), –122.5 (m, 4F, $2CF_2$), –123.3 (m, 4F, $2CF_2$), –125.9 (m, 4F, $2CF_2$).

1,2,4-($R_{f8}CH_2CH_2)_2C_6H_3ICl_2$ [s9]**.** A round bottom flask was charged with 1,2,4-(R_{f8}-$CH_2CH_2)_2C_6H_3I$ (0.42 g, 0.38 mmol) and perfluoroheptane (5 mL). The suspension was mildly heated to give a colorless solution, and H_2O (5 mL) and aqueous NaOCl (2.5% w/w, 2 mL) were added with stirring to give a biphasic mixture. Then conc. HCl (1 mL) was slowly added with vigorous stirring. After 5 min, the white precipitate was isolated by filtration (glass frit) and dried by means of an air flow (0.5 h) to give a monohydrate of 1,2,4-($R_{f8}CH_2CH_2)_2C_6H_3ICl_2$ as a white powder (0.400 g, 0.338 mmol, 89%), mp 53-54 °C (capillary). Anal. Calcd for $C_{26}H_{11}F_{34}$-$ICl_2 \cdot H_2O$: C 26.35, H 1.11. Found: C 26.27, H 1.13. The yield of a reaction carried out in C_6F_6 (5 mL) was similar.

NMR (δ in ppm, $CDCl_3$): ^{1}H (400 MHz) 8.23 (d, $^3J_{HH}$ = 8 Hz, 1H, CH_{Ar}), 7.36 (s, 1H, CH_{Ar}), 7.20 (d, $^3J_{HH}$ = 8 Hz, 1H, CH_{Ar}), 3.41, 3.04 (2 × m, 2 × 2H, $2CH_2CF_2$), 2.65, 2.43 (2 × m, 2 × 2H, $2C\mathbf{H}_2CH_2CF_2$); $^{13}C\{^1H\}$ (100 MHz, partial) 145.8, 139.9, 137.6, 130.9, 130.6, 130.4 (6 × s, C_6H_3), 32.4, 32.1 (2 × t, $^2J_{CF}$ = 22 Hz, $2\mathbf{C}H_2CF_2$), 30.6, 26.4 (2 × s, $2\mathbf{C}H_2CH_2CF_2$); $^{19}F\{^1H\}$ (376 MHz) –80.7 (t, $^4J_{FF}$ = 8 Hz [s1-s3], 6F, $2CF_3$), –114.3 (m, 4F, $2CF_2$), –121.6 (m,

8F, $4CF_2$), –21.8 (m, 4F, $2CF_2$), –122.7 (m, 4F, $2CF_2$), –123.3 (m, 4F, $2CF_2$), –126.1 (m, 4F, $2CF_2$).

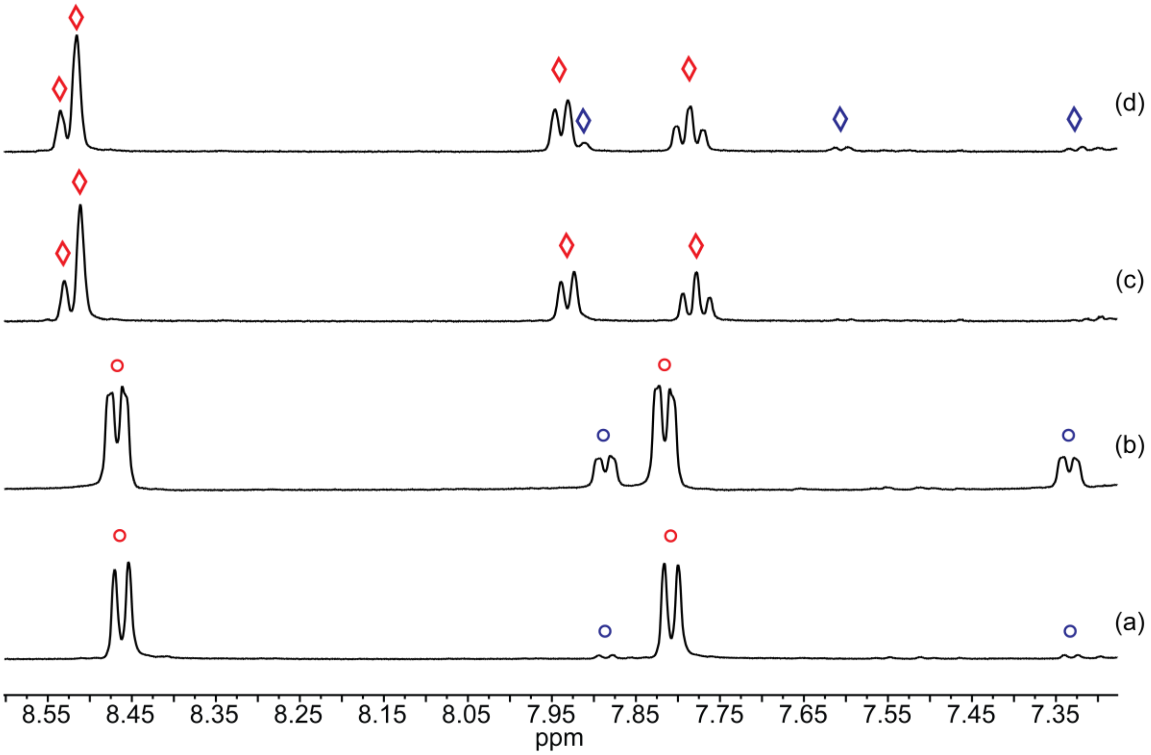

Figure S1. Partial ^{1}H NMR spectra (sp^2 CH, 500 MHz, 1:1 v/v $CDCl_3/C_6F_6$, Ph_2SiMe_2 internal standard) illustrating the stabilities of 1,4- and 1,3-$R_{f6}C_6H_4ICl_2$: (a) 1,4-$R_{f6}C_6H_4ICl_2$ shortly after dissolution (95:05 $ArICl_2$/ArI; red ° = $ArICl_2$, blue ° = ArI); (b) the preceding sample after 60 h; (c) 1,3-$R_{f6}C_6H_4ICl_2$ shortly after dissolution; (d) the preceding sample after 60 h (93:07 $ArICl_2$/ArI; red ◊ = $ArICl_2$, blue ◊ = ArI).

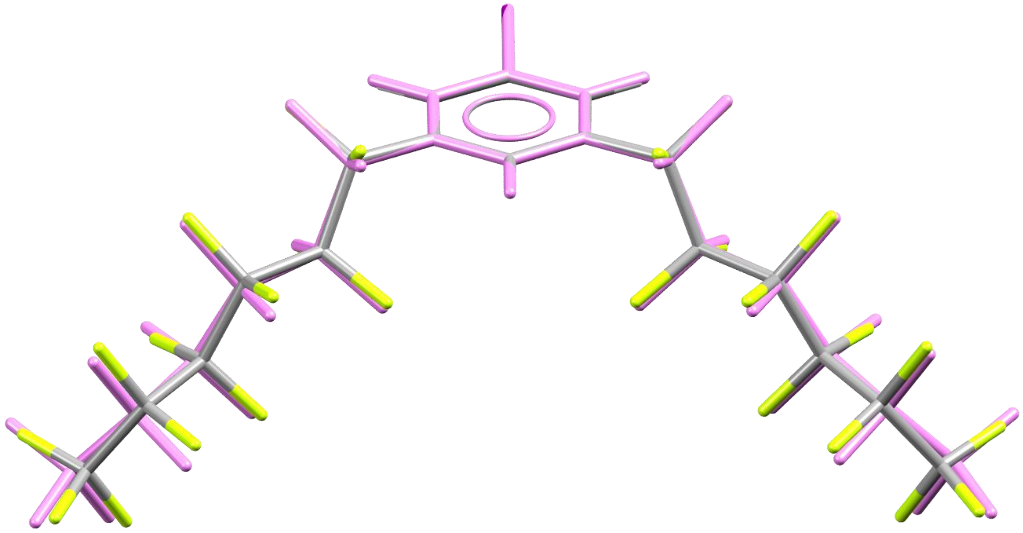

Figure S2. Overlay of the crystal structure of 1,3,5-$(R_{f6})_2C_6H_3I$ (Figure 2) with the calculated geometry (DFT, magenta).

Table S1. Calculated gas phase free energies of chlorination for selected alkyl and aryl iodides.

RI (g) or ArI (g) + Cl_2 (g) → $RICl_2$ (g) or $ArICl_2$ (g)

**Compound**	ΔG, kcal/mol
$R_{f1}I$	5.43
$R_{f2}I$	5.75
$R_{f3}I$	5.57
$R_{f4}I$	5.36
$R_{f5}I$	5.31
$R_{f6}I$	5.30
$R_{f7}I$	5.29
$R_{f8}I$	5.28
$R_{f1}CH_2I$	–0.72
$R_{f2}CH_2I$	–0.55
$R_{f3}CH_2I$	–0.51
$R_{f4}CH_2I$	–0.53
$R_{f5}CH_2I$	–0.55
$R_{f6}CH_2I$	–0.54
$R_{f7}CH_2I$	–0.56
$R_{f8}CH_2I$	–0.59
$CF_3CF_2CF_2O(CF(CF_3)CF_2O)_1CF(CF_3)CH_2I$[a]	–0.65
$CF_3CF_2CF_2O(CF(CF_3)CF_2O)_2CF(CF_3)CH_2I$[a]	–0.71
$CF_3CF_2CF_2O(CF(CF_3)CF_2O)_3CF(CF_3)CH_2I$[a]	–0.84
1,2,3,5-$CH_3(NO_2)_2C_6H_2I$[b] (**I**-Me)	0.15
1,3,5-$(NO_2)_2C_6H_3I$ (**I**)	0.88
C_6H_5I	–3.86
1,3-$R_{f6}C_6H_4I$	–1.82
1,4-$R_{f6}C_6H_4I$	–1.92
1,3,5-$(R_{f6})_2C_6H_3I$	–0.68
1,2,4-$(R_{f6}CH_2CH_2)_2C_6H_3I$[b]	–2.88
1,2,4-$(R_{f6}CH_2CH_2CH_2)_2C_6H_3I$[b] (**VII**)	–3.40
1,3,4-$(R_{f6}CH_2CH_2CH_2)_2C_6H_3I$[b] (**VIII**)	–3.75
1,2,4,6-$(R_{f6}CH_2CH_2CH_2)_3C_6H_3I$[b] (**IX**)	–4.15
$(R_{f6})_3CCH_2I$ (**X** with n = 6)	3.36
1,4-$(R_{f6})_3CC_6H_4I$ (**XII** with n = 6)	–1.93
1,4-$(R_{f8})_3CC_6H_4I$ (**XII** with n = 8)	–1.59
1,3,4-$((R_{f6})_2C)O_2C_6H_3I$[b] (**XIII** with n = 6)	–2.43

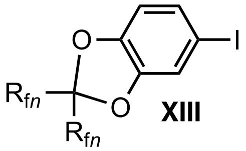

XIII

[a]Computations were conducted using the stereoisomer with S configurations at all carbon stereocenters. [b]These formulae are potentially ambiguous. The iodine atom occupies the 1 position (and the methyl group in **I**-Me the 2 position).

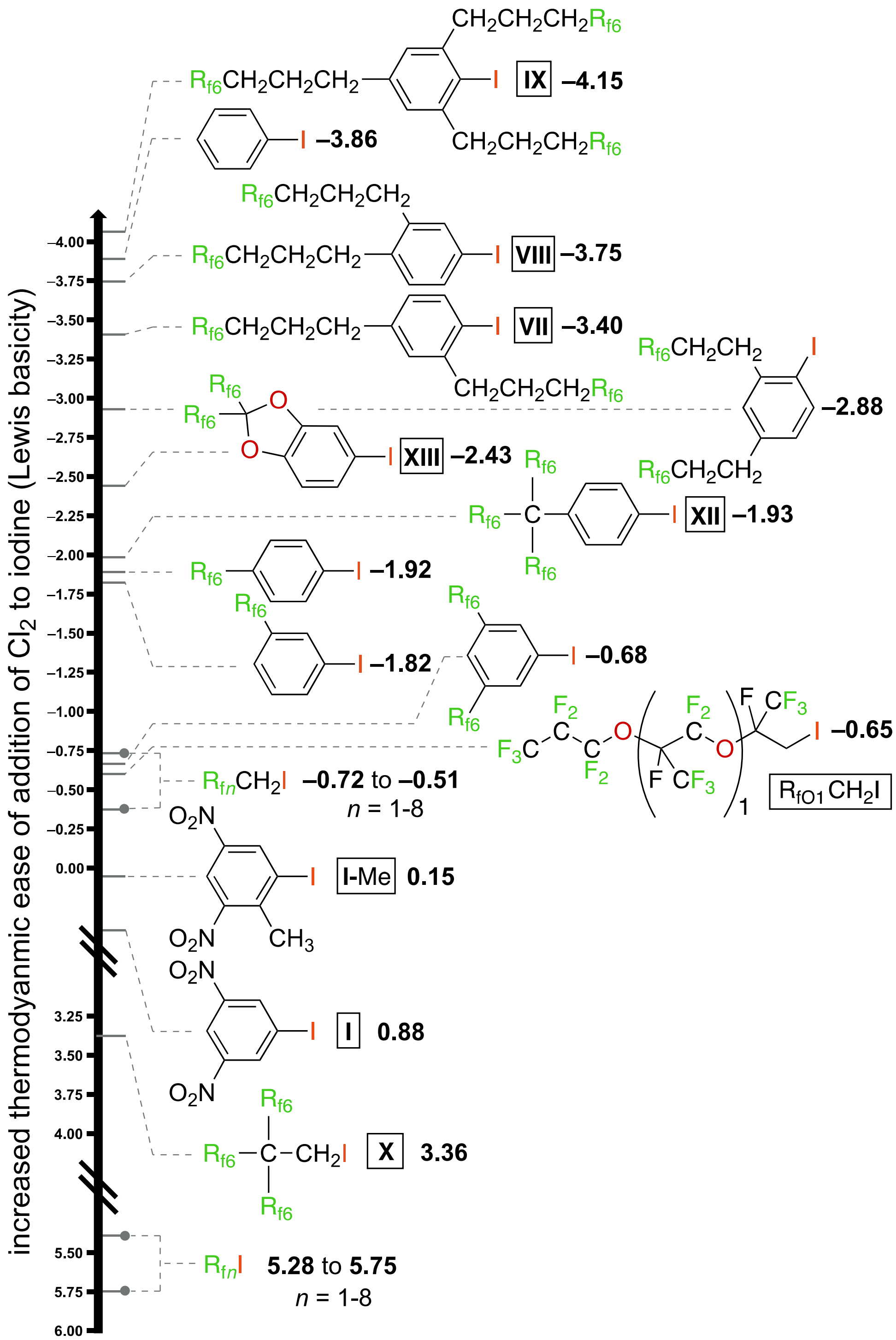

Figure S3. Free energies of chlorination of all of the types of aryl and alkyl iodides in Table S1 to the corresponding iodine(III) dichlorides in the gas phase (kcal/mol), presented in a ladder format (each iodide is more Lewis basic than that shown below it).

References

(s1) The triplets commonly observed for $CF_2CF_2C\underline{\mathbf{F}}_3$ and $CF_2CF_2C\underline{\mathbf{F}}_2X$ signals have been shown to be four bond and *not* three bond (vicinal) couplings [s2,s3].

(s2) White, H. F. *Anal. Chem.* **1966**, *38*, 625-626.

(s3) Foris, A. *Magn. Reson. Chem.* **2004**, *42*, 534-555.

(s4) These microanalytical data feature one or more values outside of normally accepted ranges but are presented nonetheless as the best fit obtained to date, and/or to illustrate a point made in the text.

(s5) The additional coupling or increased multiplicity associated with this signal as compared to the R_{fn} analog may be due to diastereomers reflecting the multiple carbon stereocenters.

(s6) This triplet may represent two overlapping doublets.

(s7) Ghosh, S. K.; Ojeda, A. S.; Guerrero-Leal, J.; Bhuvanesh, N.; Gladysz, J. A. *Inorg. Chem*. **2013**, *52*, 9369-9378.

(s8) van den Broeke, J.; Deelman, J. B.; van Koten, G. *Tetrahedron Lett*. **2001**, *42*, 8085-8087.

(s9) This formula is potentially ambiguous. For all benzenoid compounds in the paper, any iodine substituent is assigned the 1-position.

(s10) Su, H.-L.; Balogh, J.; Al-Hashimi, M.; Seapy, D. G.; Bazzi, H. S.; Gladysz, J. A. *Org. Biomol. Chem*. **2016**, 10058-10069.

(s11) These represent the two largest triplets associated with the quartet of triplets that can often be seen for the $^{13}C\{^1H\}$ signal of the terminal carbon atom of $CF_2\mathbf{C}F_3$ groups: Ribeiro, A. A. *J. Fluor. Chem.* **1997**, *83*, 61-66 (see Figure 1 therein). The presence of two inequivalent $(CF_2)_7CF_3$ groups in this molecule renders assigning exact chemical shifts to the remaining (overlapping) triplets difficult. For spectra with related complications, see the supporting information of the following paper: Richter, B.; de Wolf, E.; van Koten, G.; Deelman, B.-J. *J. Org. Chem.* **2000**, *65*, 3885-3893.
